# Morphogenesis of the T4 tail and tail fibers

**DOI:** 10.1186/1743-422X-7-355

**Published:** 2010-12-03

**Authors:** Petr G Leiman, Fumio Arisaka, Mark J van Raaij, Victor A Kostyuchenko, Anastasia A Aksyuk, Shuji Kanamaru, Michael G Rossmann

**Affiliations:** 1 Ecole Polytechnique Fédérale de Lausanne (EPFL), Institut de physique des systèmes biologiques, BSP-415, CH-1015 Lausanne, Switzerland; 2 Tokyo Institute of Technology, Graduate School of Bioscience and Biotechnology, B-9 4259 Nagatsuta, Midori-ku, Yokohama, 226-8501, Japan; 3 Centro Nacional de Biotecnologia, Dpto de Estructura de Macromoleculas, Campus Cantoblanco c/Darwin 3, E-28049 Madrid, Spain; 4 Duke-NUS Graduate Medical School, Program in Emerging Infectious Diseases, 30 Medical Drive, Brenner Centre for Molecuar Medicine, NUS, 117609, Singapore; 5 Purdue University, Department of Biological Sciences, 915 W. State Street, West Lafayette, IN 47907-2054, USA

## Abstract

Remarkable progress has been made during the past ten years in elucidating the structure of the bacteriophage T4 tail by a combination of three-dimensional image reconstruction from electron micrographs and X-ray crystallography of the components. Partial and complete structures of nine out of twenty tail structural proteins have been determined by X-ray crystallography and have been fitted into the 3D-reconstituted structure of the "extended" tail. The 3D structure of the "contracted" tail was also determined and interpreted in terms of component proteins. Given the pseudo-atomic tail structures both before and after contraction, it is now possible to understand the gross conformational change of the baseplate in terms of the change in the relative positions of the subunit proteins. These studies have explained how the conformational change of the baseplate and contraction of the tail are related to the tail's host cell recognition and membrane penetration function. On the other hand, the baseplate assembly process has been recently reexamined in detail in a precise system involving recombinant proteins (unlike the earlier studies with phage mutants). These experiments showed that the sequential association of the subunits of the baseplate wedge is based on the induced-fit upon association of each subunit. It was also found that, upon association of gp53 (gene product 53), the penultimate subunit of the wedge, six of the wedge intermediates spontaneously associate to form a baseplate-like structure in the absence of the central hub. Structure determination of the rest of the subunits and intermediate complexes and the assembly of the hub still require further study.

## Introduction

**Figure 1:**
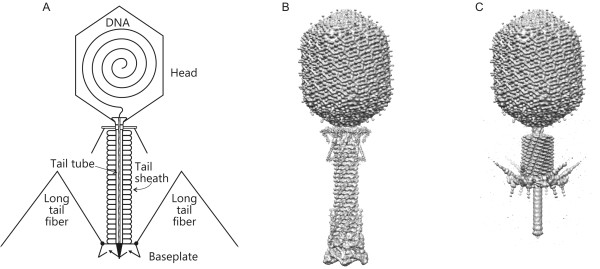
**Structure of bacteriophage T4**. (**A**) Schematic representation; CryoEM-derived model of the phage particle prior to (**B**) and upon (**C**) host cell attachment. Tail fibers are disordered in the cryoEM structures, as they represent the average of many particles each having the fibers in a slightly different conformation.

The structures of bacteriophages are unique among viruses in that most of them have tails, the specialized host cell attachment organelles. Phages that possess a tail are collectively called "Caudovirales" [1]. The family Caudovirales is divided into three sub-families according to the tail morphology: Myoviridae (long contractile tail), Siphoviridae (long non-contractile tail), and Podoviridae (short non-contractile tail). Of these, Myoviridae phages have the most complex tail structures with the greatest number of proteins involved in the tail assembly and function. Bacteriophage T4 belongs to this sub-family and has a very high efficiency of infection, likely due to its complex tails and two sets of host-cell binding fibers (Figure 1). In laboratory conditions, virtually every phage particle can adsorb onto a bacterium and is successful in injecting the DNA into the cytosol [2].

Since the emergence of conditional lethal mutants in the 1960's [3], assembly of the phage as well as its molecular genetics have been extensively studied as reviewed in "Molecular biology of bacteriophage T4" [4]. During the past ten years, remarkable progress has been made in understanding the conformational transformation of the tail baseplate from a "hexagon" to a "star" shape, which occurs upon attachment of the phage to the host cell surface. Three-dimensional image reconstructions have been determined of the baseplate, both before [5] and after [6] tail contraction using cryo-electron microscopy and complete or partial atomic structures of eight out of 15 baseplate proteins have been solved [7-14]. The atomic structures of these proteins were fitted into the reconstructions [15]. The fact that the crystal structures of the constituent proteins could be unambiguously placed in both conformations of the baseplate indicated that the gross conformational change of the baseplate is caused by a rearrangement or relative movement of the subunit proteins, rather than associated with large structural changes of individual proteins. This has now provided a good understanding of the mechanics of the structural transformation of the baseplate, which will be discussed in this review.

## Assembly Pathway of the Tail

The tail of bacteriophage T4 is a very large macromolecular complex, comprised of about 430 polypeptide chains with a molecular weight of approximately 2 × 10^7 ^(Tables 1, 2 and 3). Twenty two genes are involved in the assembly of the T4 tail (Tables 1, 2 and 3). The tail consists of a sheath, an internal tail tube and a baseplate, situated at the distal end of the tail. Two types of fibers (the long tail fibers and the short tail fibers), responsible for host cell recognition and binding, are attached to the baseplate.

**Table 1:** Tail proteins listed in the order of assembly into the complete tail 172425.

Protein	Monomer mass (kDa)	Oligomeric state in solution	Number of monomer copies in the tail	Location and remarks	Protein Data Bank accession code
gp11	23.7	Trimer	18	Wedge, STF^# ^binding interface	1EL6
gp10	66.2	Trimer	18	Wedge, STF attachment	2FKK
gp7	119.2	Monomer	6	Wedge	
gp8	38.0	Dimer	12	Wedge	1N7Z
gp6	74.4	Dimer	12	Wedge	3H2T
gp53	23.0	ND*	6	Wedge	
gp25	15.1	Dimer^$^	6	Wedge	

gp5	63.7	Trimer	3	Hub	1K28
gp27	44.4	Trimer	3	Hub	1K28
gp29	64.4	ND	3	Hub, tail tube, Tape measure	

gp28	17.3	ND	1‡	Hub; the tip of gp5 needle?	

gp9	31.0	Trimer	18	Wedge, LTF^¶ ^attachment site	1S2E
gp12	55.3	Trimer	18	Baseplate outer rim, STF	1H6W, 1OCY
gp48	39.7	ND	6	Baseplate-tail tube junction	
gp54	35.0	ND	6	Baseplate-tail tube junction	

gp19	18.5	Polymer	138	Tail tube	
gp3	19.7	Hexamer	6	Tail tube terminator	
gp18	71.2	Polymer	138	Tail sheath	3FOA
gp15	31.4	Hexamer	6	Tail terminator	

^# ^STF, short tail fiber.

* ND, not determined.

^$ ^P.G. Leiman, unpublished data.

^¶ ^LTF, long tail fiber.

‡ Copy number and presence in the tail are uncertain.

**Table 2:** Chaperones involved in the assembly of the tail, tail fibers and attachment of the fibers to the phage particle 7172343446274.

Protein	Monomer mass (kDa)	Oligomeric state in solution	Function	Protein Data Bank accession code
gp8	38.0	Dimer	Folding of gp6	1N7Z
gp26	23.9	ND*	Hub assembly chaperone	
gp51	29.3	ND	Hub assembly chaperone	
gp57A	5.7	Mixture: Trimer-Hexamer-Dodecamer	Folding of gp12, gp34, gp37	
gp38	22.3	ND	Folding of gp37	
gpwac	51.9	Trimer	LTF^¶ ^to baseplate attachment	1AA0
gp63	45.3	ND	LTF to baseplate attachment	

* ND, not determined.

^¶ ^LTF, long tail fiber.

**Table 3:** T4 fibers 17186265.

Fiber	Gene	Monomer mass (kDa)	No. of protein chains per fiber	Location
STF	12	55.3	3	Baseplate

	34	140.0	3	Proximal part, connected to the baseplate
LTF	35	30.0	1	Hinge region
	36	23.0	3	Distal part, hinge connection
	37	109.0	3	Distal part, receptor recognition tip

Head whisker	*wac*	51.9	3	Head-tail joining region

**Figure 2:**
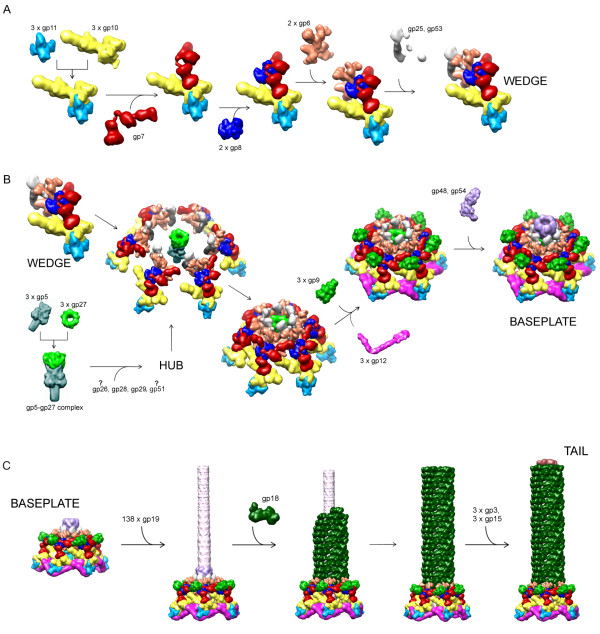
**Assembly of the tail**. Rows **A**, **B **and **C **show the assembly of the wedge; the baseplate and the tail tube with the sheath, respectively.

The assembly pathway of the T4 tail has been extensively studied by a number of authors and has been reviewed earlier [16-20]. The main part of the assembly pathway has been elucidated by Kikuchi and King [21-23] with the help of elaborate complementation assays and electron microscopy. The lysates of various amber mutant phage-infected cells were fractionated on sucrose density gradients and complemented with each other *in vitro*. The assembly pathway is strictly ordered and consists of many steps (Figure 2). If one of the gene products is missing, the assembly proceeds to the point where the missing product would be required, leaving the remaining gene products in an "assembly naïve" soluble form, as is especially apparent in the baseplate wedge assembly. The assembly pathway has been confirmed by *in vivo *assembly experiments by Ferguson and Coombs (Table 1) [24] who performed pulse-chase experiments using ^35^S-labeled methionine and monitored the accumulation of the labeled gene products in the completed tail. They confirmed the previously proposed assembly pathway and showed that the order of appearance of the labeled gene products also depended on the pool size or the existing number of the protein in the cell. The tail genes are 'late' genes that are expressed almost simultaneously at 8 to 10 min after the infection, indicating that the order of the assembly is determined by the protein interactions, but not by the order of expression.

The fully assembled baseplate is a prerequisite for the assembly of the tail tube and the sheath both of which polymerize into the extended structure using the baseplate as the assembly nucleus (Figure 2). The baseplate is comprised of about 140 polypeptide chains of at least 16 proteins. Two gene products, gp51 and gp57A, are required for assembly, but are not present in the final particle. The baseplate has sixfold symmetry and is assembled from 6 wedges and the central hub. The only known enzyme associated with the phage particle, the T4 tail lysozyme, is a baseplate component. It is encoded by gene 5 (gp5).

The assembly of the wedge, consisting of seven gene products (gp11, gp10, gp7, gp8, gp6, gp53 and gp25), is strictly ordered. When one of the gene products is missing, the intermediate complex before the missing gene product is formed and the remaining gene products stay in a free form in solution. Gp11 is an exception, which can bind to gp10 at any step of the assembly. Recently, all the intermediate complexes and the complete wedge as well as all the individual gene products of the wedge were isolated, and the interactions among the gene products were examined [25]. An unexpected finding was that gp6, gp53 and gp25 interact with each other weakly. Gp53, however, binds strongly to the precursor wedge complex only after gp6 has bound. Similarly, gp53 is required for gp25 binding. These findings strongly indicated that the strict sequential order of the wedge assembly is due to a conformational change of the intermediate complex, which results in the creation of a new binding site rather than formation of a new binding site at the interface between the newly bound gene product and the precursor complex. Another unexpected finding was that the wedge precursor complexes spontaneously assemble into sixfold symmetrical star-shaped baseplate-like, 43S structure as soon as gp53 binds. The 43S baseplate decreases its sedimentation coefficient to 40S after gp25 and gp11 binding, apparently due to a structural change in the baseplate [21-23]. Based on these findings, Yap *et al*. [25] have postulated that the 40S star-shaped particle is capable of binding the hub and the six short, gp12 tail fibers, to form the 70S dome-shaped baseplate, found in the extended tail.

Several groups studied the assembly and composition of the central part of the baseplate - the hub - and arrived at different, rather contradictory, conclusions [17]. The assembly of the hub is complicated by a branching pathway and by the presence of gp51, an essential protein of unknown function [26]. Structural studies suggest that the hub consists of at least four proteins: gp5, gp27, gp29 and another unidentified small protein, possibly, gp28 [5]. Recent genetic studies support some of the earlier findings that the hub contains gp26 and gp28 [27].

After the formation of the 70S dome-shaped baseplate containing the short tail fibers, six gp9 trimers (the "socket proteins" of the long tail fibers) bind to the baseplate. Gp48 and gp54 bind to the 'upper' part of the baseplate dome to form the platform for polymerization of gp19 for formation of the tube.

The detailed mechanism of the length determination of the tube is unknown, but the strongest current hypothesis suggests that gp29 is incorporated into the baseplate in an unfolded form. Gp29, the "tape-measure protein", extends as more and more copies of the tail tube protomer, gp19, are added to the growing tube[28]. At the end of the tube, the capping protein, gp3, binds to the last row of gp19 subunits (and, possibly, to gp29) to stabilize them. The tail sheath is built from gp18 subunits simultaneously as the tube, using the tube as a scaffold. When the sheath reaches the length of the tube, the tail terminator protein, gp15, binds to gp3 and the last row of gp18 subunits, completing the tail, which becomes competent for attachment to the head. Both gp15 and gp3 form hexameric rings [29].

The assembly pathway of the tail is a component of **Movie 1 **(http://www.seyet.com/t4_virology.html), which describes the assembly of the entire phage particle.

## Tail Structure

### Structure of the baseplate and its constituent proteins

The tail consists of the sheath, the internal tail tube and the baseplate, situated at the distal end of the tail (Figures 1 and 2). During attachment to the host cell surface, the tail undergoes a large conformational change: The baseplate opens up like a flower, the sheath contracts, and the internal tube is pushed through the baseplate, penetrating the host envelope. The phage DNA is then released into the host cell cytoplasm through the tube. The tail can, therefore, be compared to a syringe, which is powered by the extended spring, the sheath, making the term "macromolecular nanomachine" appropriate.

The baseplate conformation is coupled to that of the sheath: the "hexagonal" conformation is associated with the extended sheath, whereas the "star" conformation is associated with the contracted sheath that occurs in the T4 particle after attachment to the host cell. Before discussing more fully the baseplate and tail structures in their two conformations, the crystal structures of the baseplate constituent proteins as well as relevant biochemical and genetic data will be described.

### Crystal structure of the cell-puncturing device, the gp5-gp27 complex

**Figure 3:**
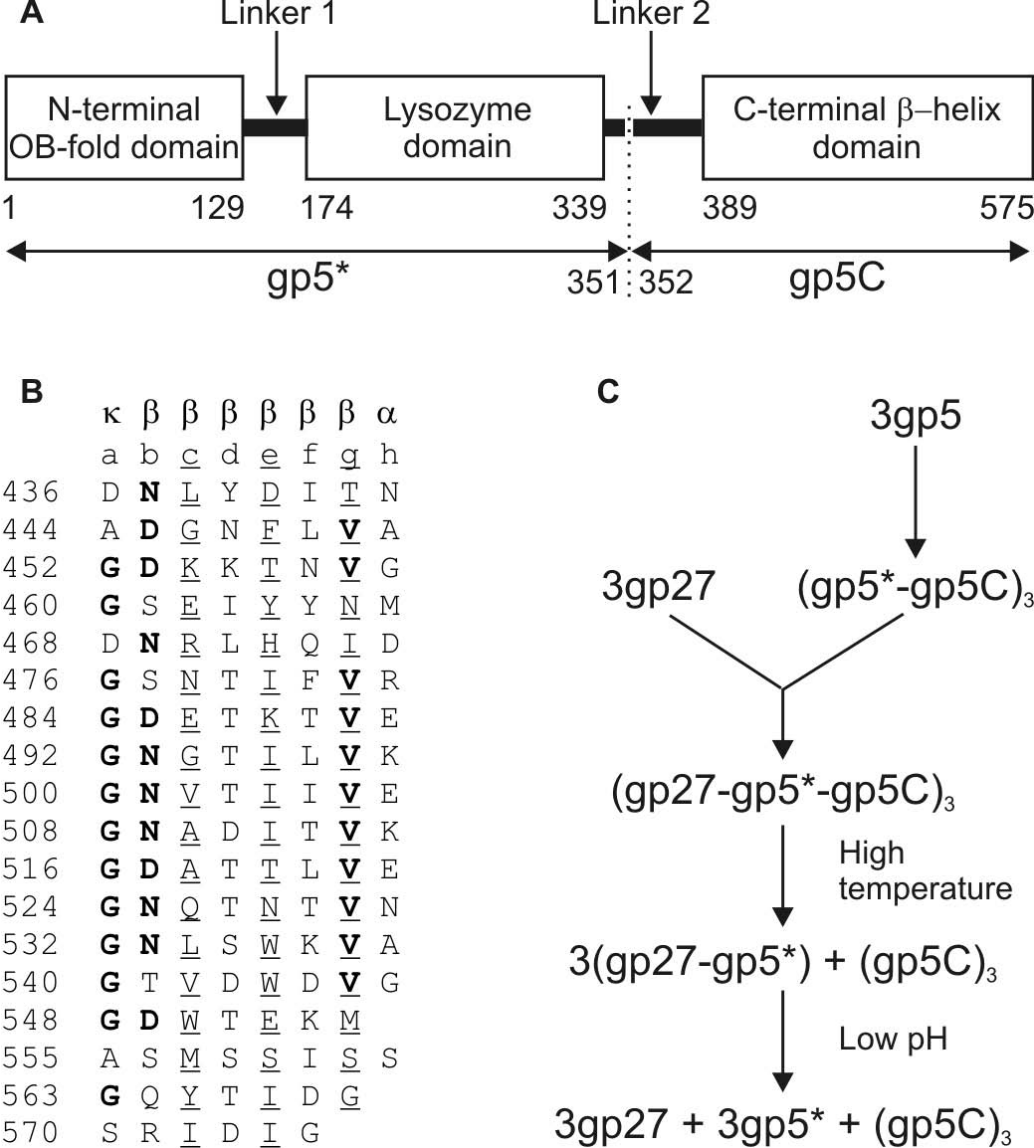
Assembly of (gp27-gp5*-gp5C)_**3**_**; reprinted from **[13]. **A**, Domain organization of gp5. The maturation cleavage is indicated with the dotted line. Initial and final residue numbers are shown for each domain. **B**, Alignment of the octapeptide units composing the intertwined part of the C-terminal β-helix domain of gp5. Conserved residues are in bold print; residues facing the inside are underlined. The main chain dihedral angle configuration of each residue in the octapeptide is indicated at the top by κ (kink), β (sheet), and α (helix). **C **Assembly of gp5 and gp27 into the hub and needle of the baseplate.

Gp5 was identified as the tail-associated lysozyme, required during infection but not for cell lysis [30]. The lysozyme domain of gp5 is the middle part of the gp5 polypeptide [31]. It has 43% sequence identity to the cytoplasmic T4 lysozyme, encoded by gene e and called T4L [32]. Gp5 was found to undergo post-translational proteolysis [31], which was believed to be required for activation. Kanamaru *et al*. [33] showed that the C-terminal domain of gp5, which they named gp5C, is a structural component of the phage particle. Furthermore, Kanamaru *et al*. [33] reported that 1) gp5C is an SDS- and urea-resistant trimer; 2) gp5C is responsible for trimerization of the entire gp5; 3) gp5C is rich in β-structure; 4) post-translational proteolysis occurs between Ser351 and Ala352; 5) gp5C dissociates from the N-terminal part, called gp5*, at elevated temperatures; and that 6) the lysozyme activity of the trimeric gp5 in the presence of gp5C is only 10% of that of the monomeric gp5*. The amino acid sequence of gp5C contains eleven **V**X**G**XXXXX repeats. Subsequent studies showed that gp5 forms a stable complex with gp27 in equimolar quantities and that this complex falls apart in low pH conditions (Figure 3). Upon cleavage of gp5, this complex consists of 9 polypeptide chains, represented as (gp27-gp5*-gp5C)_3_.

**Figure 4:**
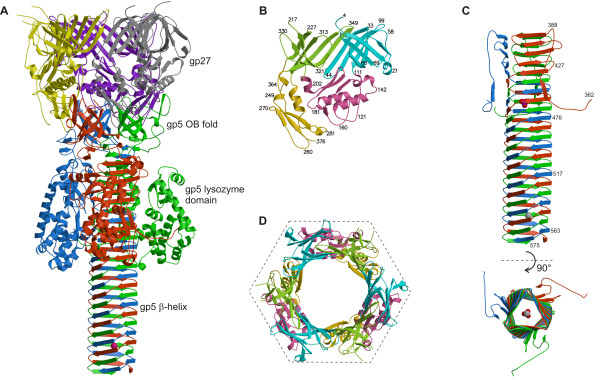
**Structure of the gp5-gp27 complex**. **A**, The gp5-gp27 trimer is shown as a ribbon diagram in which each chain is shown in a different color. **B**, Domains of gp27. The two homologous domains are colored in light green and cyan. **C**, Side and end on views of the C-terminal β-helical domain of gp5. **D**, The pseudohexameric feature of the gp27 trimer is outlined with a hexamer (domains are colored as in **B**).

The crystal structure of the gp5-gp27 complex was determined to a resolution of 2.9 Å [13]. The structure resembles a 190 Å long torch (or flashlight) (Figure 4) with the gp27 trimer forming the cylindrical "head" part of the structure. This hollow cylinder has internal and external diameters of about 30 Å and 80 Å, respectively, and is about 60 Å long. The cylinder encompasses three N-terminal domains of the trimeric gp5* to which the 'handle' of the torch is attached. The 'handle' is formed by three intertwined polypeptide chains constituting the gp5 C-terminal domain folded into a trimeric β-helix. The three gp5 lysozyme domains are adjacent to the β-helix. Two long peptide linkers run along the side of the β-helix, connecting the lysozyme domain with the gp5 N- and C-terminal domains. The linker joining the lysozyme domain to the β-helix contains the cleavage site between gp5* and gp5C.

Two domains of gp27 (residues 2 to 111 and residues 207-239 plus 307-368) are homologous (Figure 4). They have similar seven- or eight-stranded, antiparallel β-barrel structures, which can be superimposed on each other with the root mean square deviation (RMSD) of 2.4 Å between the 63 equivalent C_α _atoms, representing 82% of all C_α _atoms. The superposition transformation involves an approximately 60° rotation about the crystallographic threefold axis. Thus, these domains of gp27 form a pseudo-sixfold-symmetric torus in the trimer, which serves as the symmetry adjuster between the trimeric gp5-gp27 complex and the sixfold-symmetric baseplate. Notwithstanding the structural similarity of these two domains, there is only 4% sequence identity of the structurally equivalent amino acids in these two domains. Nevertheless, the electrostatic charge distribution and hydrophilic properties of the gp27 trimer are roughly sixfold symmetric.

Gp5* consists of the N-terminal OB-fold domain and the lysozyme domain. The OB-fold domain is a five-stranded antiparallel β-barrel with a Greek-key topology that was originally observed as being an oligosaccharide/oligonucleotide-binding domain [34]. It is clear now that this fold shows considerable variability of its binding specificity, although the substrate binding site location on the surfaces on most OB-folds has a common site [35]. It is unlikely that the gp5 N-terminal domain is involved in polysaccharide binding, as it lacks the polar residues required for binding sugars. Most probably, the OB-fold has adapted to serve as an adapter between the gp27 trimer and the C-terminal β-helical domain.

The structure of the gp5 lysozyme domain is similar to that of hen egg white lysozyme (HEWL) and T4L having 43% sequence identity with the latter. The two T4 lysozyme structures can be superimposed with an RSMD of 1.1 Å using all C_α _atoms in the alignment. There are two small additional loops in gp5, constituting a total of 5 extra residues (Val211-Arg212 and Asn232-Pro233,-Gly234). The active site residues of HEWL, T4L and gp5 are conserved. The known catalytic residues of T4L, Glu11, Asp20, and Thr26, correspond to Glu184, Asp193, and Thr199 in gp5, respectively, establishing that the enzymatic mechanism is the same and that the gp5 lysozyme domain, T4L and HEWL have a common evolutionary origin.

By comparing the crystal structure of T4L with bound substrate [36] to gp5, the inhibition of gp5 lysozyme activity in the presence of the C-terminal β-helix can be explained. Both gp5 and T4L have the same natural substrate, namely *E. coli *periplasmic cell wall, the major component of which ((NAG-NAM)-LAla-D*iso*Glu-DAP-DAla [36] ) contains sugar and peptide moieties. In the gp5 trimer, the linker connecting the lysozyme domain to the β-helix prevents binding of the peptide portion of the substrate to the lysozyme domain. At the same time, the polysaccharide binding cleft is sterically blocked by the gp5 β-helix. Dissociation of the β-helix removes both of these blockages and restores the full lysozyme activity of gp5*.

Gp5C, the C-terminal domain of gp5, is a triple-stranded β-helix (Figure 4). Three polypeptide chains wind around each other to create an equilateral triangular prism, which is 110 Å long and 28 Å in diameter. Each face has a slight left-handed twist (about 3° per β-strand), as is normally observed in β-sheets. The width of the prism face tapers gradually from 33 Å at the amino end to 25 Å at the carboxy end of the β-helix, thus creating a pointed needle. This narrowing is caused by a decrease in size of the external side chains and by the internal methionines 554 and 557, which break the octapeptide repeat near the tip of the helix. The first 5 β-strands (residues 389-435) form an antiparallel β-sheet, which forms one of the three faces of the prism. The succeeding 18 β-strands comprise a 3-start intertwined β-helix together with the other two, threefold-related polypeptides. The intertwined C-terminal part of the β-helical prism (residues 436-575) is a remarkably smooth continuation of its three non-intertwined N-terminal parts (residues 389-435).

The octapeptide sequence of the helical intertwined part of the prism (residues **a **through **h**) has dominant glycines at position **a**, asparagines or aspartic acids at position **b**, valines at position **g**, and polar or charged residues at position **h**. Residues **b **through **g **form extended β-strands (Ramachandran angles φ ≈ -129°, ψ ≈ 128°) that run at an angle of 75° with respect to the helix axis. The glycines at position **a **(φ = -85°, ψ = -143°, an allowed region of the Ramachandran diagram) and residues at position **h **(φ = -70°, ψ = -30°, typical for α-helices) kink the polypeptide chain by about 130° clockwise. The conserved valines at position **g **always point to the inside of the β-helix and form a "knob-into-holes" arrangement with the main chain atoms of the glycines at position **a **and the aliphatic part of the side chains of residues at position **c**. Asp436 replaces the normal glycine in position **a **and is at the start of the β-helix. This substitution may be required for folding of the β-helix, because the Asp436 O_δ _atom makes a hydrogen bond with O_γ _of Ser427 from the threefold-related polypeptide chain. The side chain oxygen atoms of Asp468, which also occupies position **a**, forms hydrogen bonds with residues in the lysozyme domain.

The interior of the β-helix is progressively more hydrophobic toward its C-terminal tip. The middle part of the helix has a pore, which is filled with water molecules bound to polar and charged side chains. The helix is stabilized by two ions situated on its symmetry axis: an anion (possibly, a phosphate) coordinated by three Lys454 residues and a hydrated Ca^2+ ^cation (S. Buth, S. Budko, P. Leiman unpublished data) coordinated by three Glu552 residues. These features contribute to the chemical stability of the β-helix, which is resistant to 10% SDS and 2 M guanidine HCl. The surface of the β-helix is highly negatively charged. This charge may be necessary to repel the phosphates of the lipid bilayer when the β-helix penetrates through the outer cell membrane during infection.

### Crystal structures of gp6, gp8, gp9, gp10, gp11 and gp12

**Figure 5:**
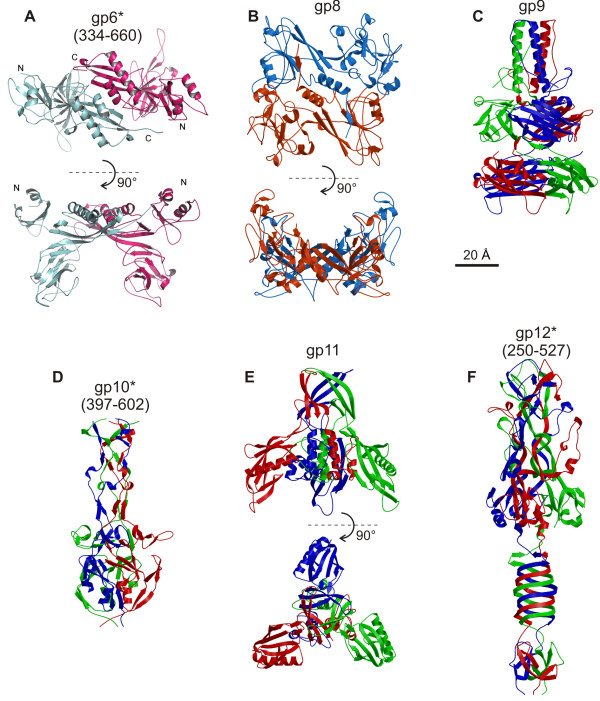
**Crystal structures of the baseplate proteins**. The star (*) symbol after the protein name denotes that the crystal structure is available for the C-terminal fragment of the protein. Residue numbers comprising the solved structure are given in parentheses.

Genes of all the T4 baseplate proteins were cloned into high level expression vectors individually and in various combinations. Proteins comprising the periphery of the baseplate showed better solubility and could be purified in amounts sufficient for crystallization. The activity was checked in complementation assays using a corresponding amber mutant phage. It was possible to crystallize and solve structures of the full-length gp8, gp9 and gp11 (Figure 5) [8-10]. The putative domain organization of gp10 was derived from the cryoEM map of the baseplate. This information was used to design a deletion mutant constituting the C-terminal domain, which was then crystallized [11]. A stable deletion mutant of gp6 suitable for crystallization was identified using limited proteolysis (Figure 5) [7]. Full-length gp12 showed a very high tendency to aggregation. Gp12 was subjected to limited proteolysis in various buffers and conditions. Two slightly different proteolysis products, which resulted from these experiments, were crystallized (Figure 5) [12,14]. Due to crystal disorder, it was possible to build an atomic model for less than half of the crystallized gp12 fragments [12,14].

Two proteins, gp6 and gp8, are dimers, whereas the rest of the crystallized proteins - gp9, gp10, gp11 and gp12 - are trimers. None of the proteins had a structural homolog in the Protein Data Bank when these structures were determined. Neither previous studies nor new structural information suggested any enzymatic activity for these proteins. The overall fold of gp12 is the most remarkable of the six mentioned proteins. The topology of the C-terminal globular part is so complex that it creates an impression that the three polypeptide chains knot around each other [14]. This is not the case, however, because the polypeptide chains can be pulled apart from their ends without entanglement. Thus the fold has been characterized as being 'knitted', but not 'knotted' [14]. Gp12 was reported to be a Zn-containing protein [37] and X-ray fluorescent data supported this finding, although Zn was present in the purification buffer [14]. The Zn atom was found to be buried deep inside the C-terminal domain. It is positioned on the threefold axis of the protein and is coordinated by the side chains of His445 and His447 from each of the three chains, resulting in octahedral geometry that is unusual for Zn [12,14,38].

Although gp12, like gp5, contains a triple-stranded β-helix (Figure 5) these helices are quite different in their structural and biochemical properties. The gp12 β-helix is narrower than the gp5 β-helix because there are 6 residues (on average) per turn in the gp12 β-helix compared to 8 in gp5. The interior of the gp12 β-helix is hydrophobic, whereas only the interior of the C-terminal tip of the gp5 β-helix is hydrophobic, but the rest is quite hydrophilic, contains water, phosphate and lipid molecules (S. Buth, S. Budko, P. Leiman unpublished data). Furthermore, the gp12 β-helix lacks the well defined gp5-like repeat.

Many functional analogs of the T4 short tail fibers in other bacteriophages have enzymatic activity and are called tailspikes. The endosialidase from phage K1F and its close homologs from phages K1E, K1-5 and CUS3 contain a very similar β-helix that has several small loops, which create a secondary substrate-binding site [39-41]. The gp12-like β-helix can be found in tail fibers of many lactophages [42], and is a very common motif for proteins that participate in lipopolysaccharide (LPS) binding. However, most gp12-like β-helices do not possess LPS binding sites. Furthermore, unlike gp5, the gp12-like β-helix cannot fold on its own, requiring a chaperone, (e.g. T4 gp57A) for folding correctly [43,44]. Nevertheless, gp12-like β-helix might have enough flexibility and possesses other properties that render give it LPS binding proteins.

The T4 baseplate is significantly more complex than that of phage P2 or Mu, two other well studied contractile tail phages [45,46], and contains at least five extra proteins (gp7, gp8, gp9, gp10 and gp11), all positioned at the baseplate's periphery. T4 gp25 and gp6 have genes W and J as homologs in P2, respectively ([45] and P. Leiman unpublished data). However, the origin and evolutionary relationships for the rest of the baseplate proteins cannot be detected at the amino acid level. The crystal structure of the C-terminal fragment (residues 397 - 602) of gp10 has provided some clues to understanding the evolution of T4 baseplate proteins [11].

**Figure 6:**
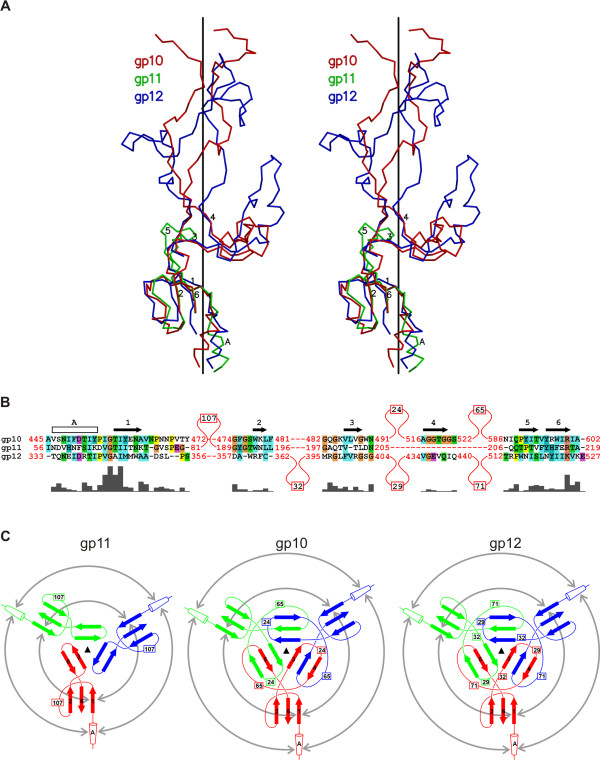
**Comparison of gp10 with other baseplate proteins; reprinted from **[11]. **A**, Stereo view of the superposition of gp10, gp11, and gp12. For clarity, the finger domain of gp11 and the insertion loop between β-strands 2 and 3 of gp12 are not shown. The β-strands are numbered 1 through 6 and the α-helix is indicated by "A". **B**, The structure-based sequence alignment of the common flower motifs of gp10, gp11, and gp12. The secondary structure elements are indicated above the sequences. The insertions between the common secondary structure elements are indicated with the number of inserted residues. The residues and their similarity are highlighted using the color scheme of the CLUSTAL program [89]. The alignment similarity profile, calculated by CLUSTAL, is shown below the sequences. **C**, The topology diagrams of the flower motif in gp10, gp11, and gp12. The circular arrows indicate interacting components within each trimer. The monomers are colored red, green, and blue. The numbers indicate the size of the insertions not represented in the diagram.

The structures of gp10, gp11 and gp12 can be superimposed onto each other (Figure 5) suggesting that the three proteins have evolved from a common primordial fold, consisting of an α-helix, a three-stranded β-sheet almost perpendicular to the helix, and an additional 2 or 3 stranded β-sheet further away from the helix (Figure 6). This structural motif is decorated by big loops inserted in various regions of the core fold, thus obscuring visual comparison. It is of significance that the three proteins are translated from the same polycistronic mRNA and are sequential in the genome. Furthermore, all three proteins are on the periphery of the baseplate and interact with each other. Apparently, over the course of the T4 evolution, these proteins have become more functionally specialized and have acquired or discarded subdomains that define the functions of the present proteins.

In addition to its structural role in the baseplate, gp8 functions as a chaperone for folding of gp6 (Table 2), which is insoluble unless co-expressed with gp8 [7]. Although wild type gp6 could not be crystallized, the structure of a gp6 mutant, constituting the C-terminal part of the protein (residues 334 - 660) has been determined [7]. The structure is a dimer, which fits well into the cryoEM map of both, the hexagonal and star-shaped baseplates [7].

### Structure of the baseplate in the hexagonal conformation

**Figure 7:**
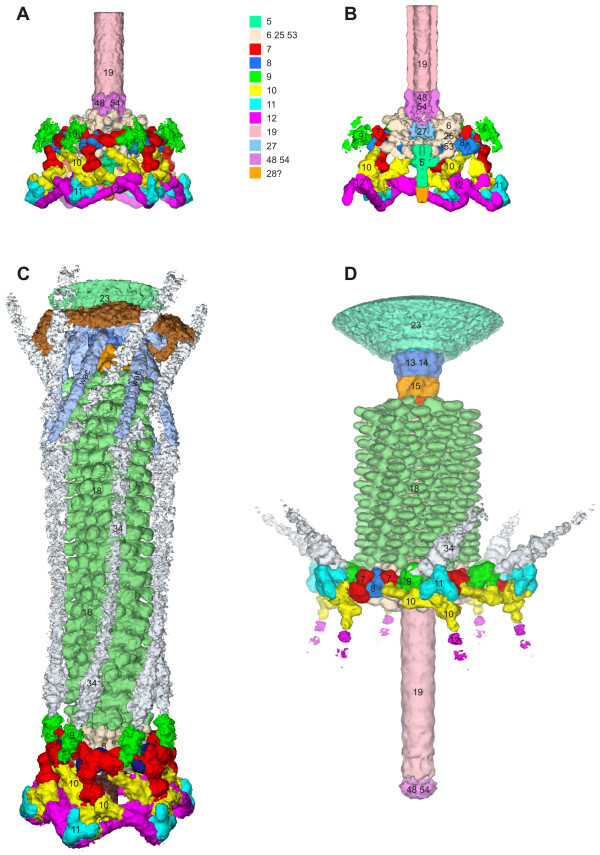
**CryoEM reconstructions of the T4 tube-baseplate complex (**A, B**) and the tail in the extended (**C**) and contracted (**D**) conformation**. Constituent proteins are shown in different colors and identified with the corresponding gene names. reprinted from [5,47] and [6].

The structure of the baseplate in the hexagonal conformation was studied both by using a phage mutant that produces the baseplate-tail tube complex (a g18¯/g23¯ double mutant), as well as by using wild type phage [5,47]. The star conformation was examined by treating the phage with 3 M urea in a neutral pH buffer [6] causing the tail to contract, but retaining the DNA in the head. This particle mimics the phage after it has attached to the host cell surface. Three-dimensional cryoEM maps of the baseplate and the entire tail in either conformation were calculated at resolutions of 12 Å and 17 Å, respectively (Figure 7). The available crystal structures were fitted into these maps.

The hexagonal baseplate is a dome-like structure with a diameter of about 520 Å around its base and about 270 Å in height. Overall, the structure resembles a pile of logs because its periphery is composed of fibrous proteins. The gp5-gp27 complex forms the central hub of the baseplate (Figure 7B). The complex serves as a coaxial continuation of the tail tube. Gp48 and/or gp54 are positioned between the gp27 trimer and the tail tube, comprised of gp19. The gp5 β-helix forms the central needle that runs along the dome's axis. A small protein with a MW of ~23 kDa is associated with the tip of the gp5 β-helix (Figure 7B). The identity of this protein is unclear, but the mass estimate suggests that it could be gp28. The tape measure protein, gp29, is almost completely disordered in the baseplate-tail tube structure. It is unclear whether gp29 degrades during the sample preparation or its structure does not agree with the sixfold symmetry assumed in generating the cryoEM map.

The earlier cross-linking and immuno-staining analysis of interactions between the baseplate wedge proteins turned out to be in good agreement with the later cryoEM results [48-50]. This is impressive considering the limitations of the techniques employed in the earlier studies. In agreement with the earlier findings, the new high resolution data show that gp10, gp11 and gp12 (the short tail fibers) constitute a major part of the baseplate's periphery. Gp9, the long tail fiber attachment protein, is also on the periphery, but in the upper part of the baseplate dome. Gp8 is positioned slightly inwards in the upper part of the baseplate dome and interacts with gp10, gp7 and gp6. The excellent agreement between the crystallographic and EM data resulted in the unambiguous locating of most of the proteins in the baseplate.

**Figure 8:**
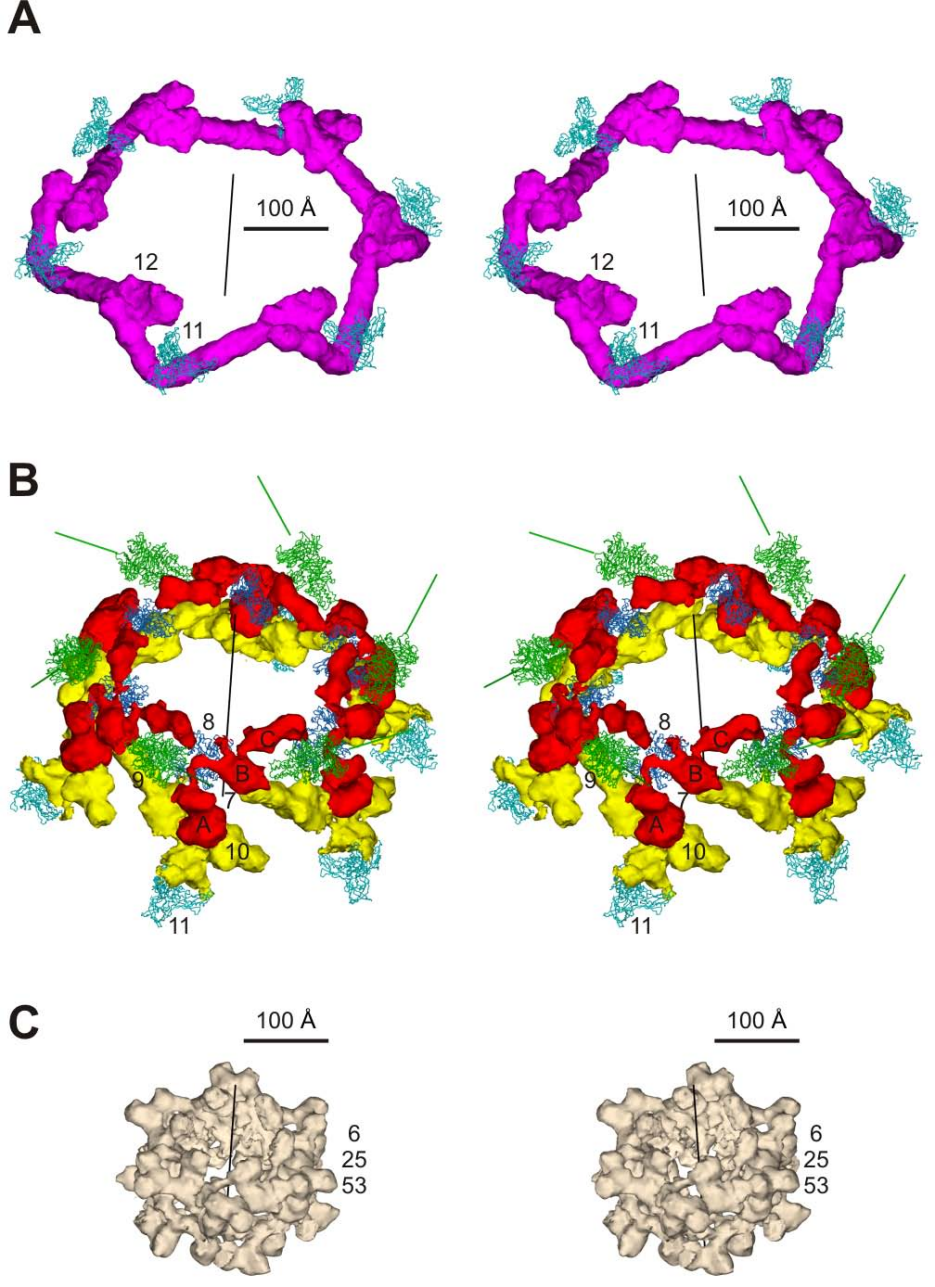
**Details of the T4 baseplate structure; reprinted from **[5]. Proteins are labeled with their respective gene numbers. **A**, The garland of short tail fibers gp12 (magenta) with gp11 structures (light blue C_α _trace) at the kinks of the gp12 fibers. The six-fold axis of the baseplate is shown as a black line. **B**, The baseplate "pins", composed of gp7 (red), gp8 (dark blue C_α _trace), gp10 (yellow), and gp11(light blue C_α _trace). Shown also is gp9 (green C_α _trace), the long tail fiber attachment protein, with a green line along its three-fold axis, representing the direction of the long tail fibers. **C**, Gp6, gp25, and gp53 density.

Six short tail fibers comprise the outermost rim of the baseplate. They form a head-to-tail garland, running clockwise if viewed from the tail towards the head (Figure 8). The N-terminus of gp12 binds coaxially to the N-terminal domain of the gp10 trimer, and the C terminus of one gp12 molecules interacts with N terminus of the neighboring molecule. The fiber is kinked at about its center, changing its direction by about 90°, as it bends around gp11. The C-terminal receptor-binding domain of gp12 is 'tucked under' the baseplate and is protected from the environment. The garland arrangement controls the unraveling of the short tail fibers, which must occur on attachment to the host cell surface.

Gp10 and gp7 consist of three separate domains each, connected by linkers (Figure 8B). Gp7 is a monomer, and it is likely that each of its domains (labeled A, B and C in Figure 8B) is a compact structure formed by a single polypeptide chain. Gp10, however, is a trimer, in which the three chains are likely to run in parallel and each of the cryoEM densities assigned to gp10 domains is threefold symmetric. The angles between the threefold axes of these domains are close to 60°. This is confirmed by the fact that the trimeric gp10_397C crystal structure fits accurately into one of the three domains assigned to gp10. At the boundary of each domain, the three gp10 chains come close together thus creating a narrowing. Interestingly, the arrangement of gp10 domains is maintained in both conformations of the baseplate suggesting that these narrow junctions are not flexible. A total of 23% of the residues in the N-terminal 200 residues of gp10 are identical and 44% of the residues have conservative substitutions when compared to the N-terminal and middle domains of T4 gp9. A homology model of the N-terminal part of gp10 agrees reasonably well with the cryoEM density assigned to the gp10 N-terminal domain. The threefold axis of this domain in the cryoEM density coincides with that of the N-terminal part of gp12, which is attached to it. The middle domain of gp10 is clamped between the three finger domains of gp11.

**Figure 9:**
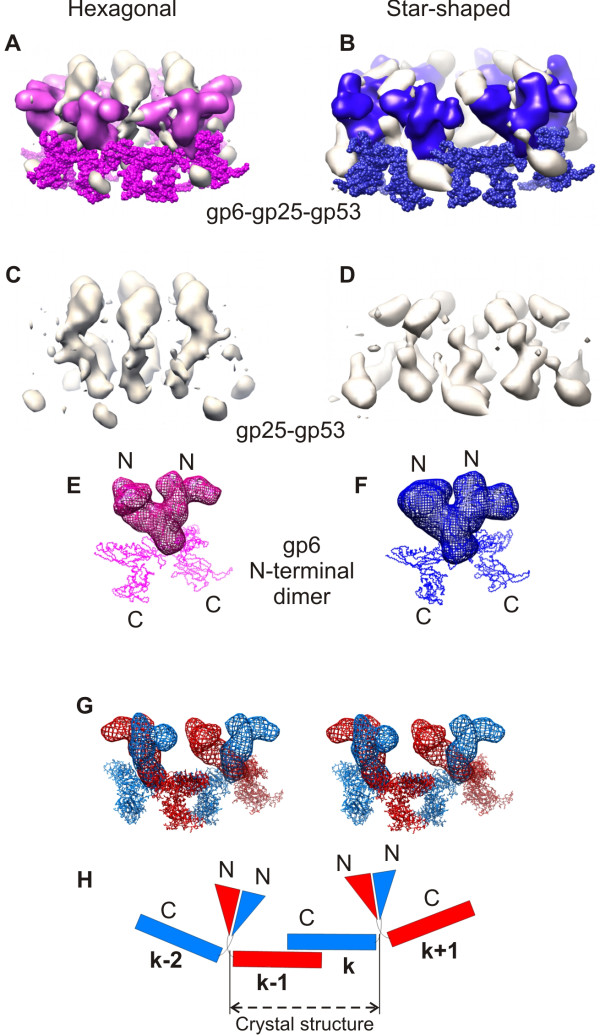
**Arrangement of gp6, gp25 and gp53 in the baseplate; reprinted from **[7]. **A, B**, Gp6 is shown in magenta for the "hexagonal" dome-shaped baseplate (left) and in blue for the star-shaped baseplate (right). The C-terminal part of gp6 corresponds to the crystal structure and is shown as a Cα trace with spheres representing each residue. The N-terminal part of gp6 was segmented from the cryo-EM map. The densities corresponding to gp53 and gp25 are shown in white. **C, D**, The densities of gp53 and gp25 after the density for the whole of gp6 was zeroed out. **E**, **F**, The N-terminal gp6 dimers as found in the baseplate wedge. The C-terminal domain is shown as a Cα trace, whereas the N-terminal domain, for which the structure remains unknown, is shown as a density mesh. **G**, A stereo view of the four neighboring gp6 molecules from the two neighboring wedges of the dome-shaped baseplate. The N-terminal part of gp6 is shown as a density mesh and the C-terminal part corresponds to the crystal structure. **H**, Schematic of the four gp6 monomers using the same colors as in **G**. The N-terminal part is shown as a triangle and the C-terminal part as a rectangle.

Gp6, gp25 and gp53 form the upper part of the baseplate dome and surround the hub complex. The cryoEM map shows that the gp6 monomer is shaped like the letter S. Six gp6 dimers interdigitate and form a continuous ring constituting the backbone of the baseplate (Figures 8 and 9). Gp6 is the only protein in the baseplate, which forms a connected ring in both conformations of the baseplate. The N- and C-terminal domains of each gp6 monomer interact with two different neighboring gp6 molecules, i.e. the N terminal domain of chain 'k' interacts with the N terminal domain of chain 'k+1', whereas the C-terminal domain of chain 'k' interacts with the C terminal domain of chain 'k-1'. It is thus possible to distinguish two types of gp6 dimers, depending on whether the N or C terminal domains of the two molecules are associated (Figure 9).

As there are only two molecules of gp6 per wedge, either the N-terminal or the C-terminal dimer has to assemble first (the intra-wedge dimer) and the other dimer is formed when the wedges associate into the ring structure (the inter-wedge dimer). Mutagenesis suggests that the Cys338 residue is critical for forming the N-terminal dimer, which therefore is likely to form the intra-wedge dimer [7]. The crystal structure represents the C-terminal inter-wedge dimer [7].

This finding is further supported by the baseplate assembly pathway. During assembly of the wedge, gp6 binds only after the attachment of gp8 [23,25]. Although a dimer of gp8 and a dimer of gp6 are present in each wedge [25], in the cryoEM baseplate map a single chain of the gp6 dimer interacts with a single chain of the gp8 dimer, whereas the other chain of the same gp6 dimer interacts with gp7. Together, gp8 and gp7 form a platform for binding of the N-terminal dimer of gp6, suggesting that the N-terminal dimer forms first during the assembly of the baseplate wedge, whereas C-terminal gp6 dimers form after six wedges associate around the hub.

The structures of the baseplate in the sheath-less tail tube assembly and in the complete tail are very similar, except for the position of gp9 (Figure 7) [5,47]. The N-terminal domain of gp9 binds to one of the gp7 domains, but the rest of the structure is exposed to the solution. The long tail fibers attach coaxially to the C-terminal domain of gp9. This arrangement allows gp9 to swivel, as a rigid body, around an axis running through the N-terminal domain, allowing the long tail fiber to move. In the extended tail structure, the long tail fibers are retracted and aligned along the tail (Figure 7c), whereas the tail tube-baseplates lack the long tail fibers. Thus, in the extended tail, the gp9 trimers point along the fibers, whereas in the tube-baseplate complexes, gp9 molecules are partially disordered due to their variable position and point sideways, on average. This variation in the positioning of gp9 is required to accommodate the full range of positions (and hence motion) observed for the long tail fibers [51].

### Structure of the baseplate in the star conformation and its comparison with the hexagonal conformation

**Figure 10:**
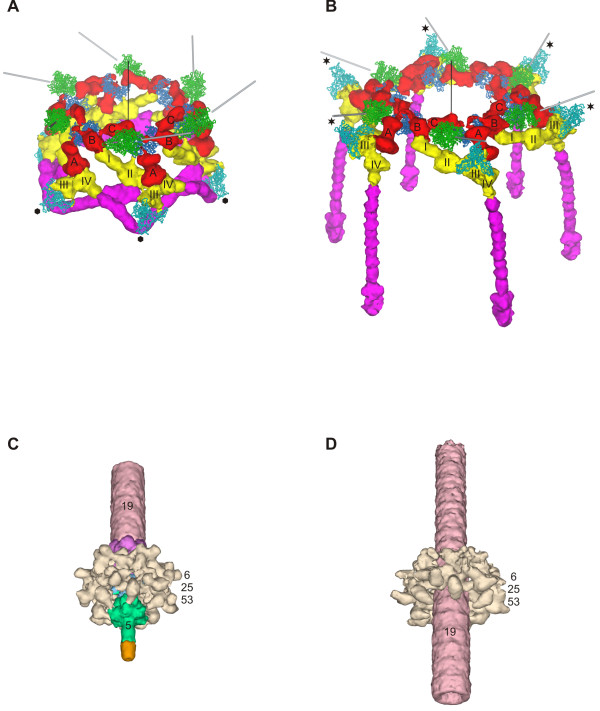
**Comparison of the baseplate in the two conformations; reprinted from **[5]. **A **and **B**, Structure of the periphery of the baseplate in the hexagonal and star conformations, respectively. Colors identify different proteins as in the other figures: gp7 (red), gp8 (blue), gp9 (green), gp10 (yellow), gp11 (cyan) and gp12 (magenta). Directions of the long tail fibers are indicated with gray rods. The three domains of gp7 are labeled with letters A, B and C. The four domains of gp10 are labeled with Roman numbers I through IV. The C-terminal domain of gp11 is labeled with a black hexagon or black star in the hexagonal or star conformations, respectively. The baseplate sixfold axis is indicated by a black line. **C **and **D**, Structure of the proteins surrounding the hub in the hexagonal and star conformations, respectively. The proteins are colored as follows: spring green, gp5; pink, gp19; sky blue, gp27; violet, putative gp48 or gp54; beige, gp6-gp25-gp53; orange, unidentified protein at the tip of gp5. A part of the tail tube is shown in both conformations for clarity.

The star-shaped baseplate has a diameter of 610 Å and is 120 Å thick along its central sixfold axis. The central hub is missing because it is pushed through and replaced by the tail tube (Figure 10). Despite large changes in the overall baseplate structure, the crystal structures and the cryoEM densities of proteins from the hexagonal baseplate can be fitted into the star shaped baseplate. This indicates that the conformational changes occur as a result of rigid body movements of the constituent proteins and/or their domains.

The largest differences between the two conformations are found at the periphery of the baseplate. In the hexagonal conformation, the C-terminal domain of gp11 points away from the phage head, and its trimer axis makes a 144° angle with respect to the six-fold axis of the baseplate (Figure 10). In the star conformation, however, the gp11 C-terminal domain points towards the phage head, and the trimer axis makes a 48° angle with respect to the baseplate sixfold axis. Thus, upon completion of the baseplate's conformational change, each gp11 molecule will have rotated by almost 100° to associate with a long, instead of a short tail fiber. The long and short tail fibers compete for the same binding site on gp11. The interaction between gp10 and gp11 is unchanged in the two conformations. As a result, the entire gp10-gp11 unit rotates by ~100° causing the N-terminal domain of gp10 to change its orientation and point towards the host cell surface (Figure 10). The short tail fiber, which is coaxially attached to the N-terminal domain of gp10, rotates and unfolds from under that baseplate and extends the C-terminal receptor-binding domain towards the potential host cell surface. In addition to the gp10-gp11 complex rotation and short tail fiber unraveling, domain A of gp7 swivels outwards by about 45° and alters its association with gp10, making the baseplate structure flat. This rearrangement brings the C-terminal domain of gp10 into the proximity of gp9 and allows the latter to interact with gp8. The structural information supports the hypothesis that the hexagonal-to-star conformational change of the baseplate is the result of a reorientation of the pins (gp7, gp10, gp11) [50] and additionally shows that the transformation also involves rearrangements of gp8, gp9, and gp12 situated around the periphery of the baseplate.

The association of gp10, gp11 and gp12 into a unit that can rotate by 100° is tight, but appears to be non-covalent. However, there could be at least one covalent bond that attaches this unit to the rest of the baseplate. Cys555, the only conserved cysteine in gp10 among all T4-like phages, is one of the residues that are involved in interactions between gp10 and domain B of gp7 in the baseplate. This cysteine might make a disulfide bond with one of eight cysteine residues in gp7, causing the gp10-gp11-gp12 complex and domain B of gp7 to act as a single rigid body during the conformational change of the baseplate. Unfortunately, residues 553-565 are disordered in the crystal structure of gp10_397C, and the exact structure of the region interacting with gp7 is uncertain. This is not surprising, as these residues might be prone to adopting various conformations, because the interaction with gp7 is not threefold symmetric.

The central part of the baseplate, which is comprised of gp6, gp25 and gp53, displays a small, but noticeable change between the two conformations of the baseplate. Both the N-terminal and C-terminal dimer contacts in the gp6 ring are maintained, but the angle between the gp6 domains changes by about 15°, accounting for the slight increase in the gp6 ring diameter (Figures 9 and 10). Therefore, the gp6 ring appears to have two functions. It is the inter-wedge 'glue', which ties the baseplate together and it is also required for maintaining the baseplate integrity during the change from hexagonal to star shaped conformations. At the same time, the gp6 ring is a framework to which the motions of other tail proteins are tied. The N-terminal domain of gp6 forms a platform onto which the first disk of the tail sheath subunits is added when the sheath it assembled. Therefore, the change in the gp6 domain orientations could be the signal that triggers the contraction of the sheath.

### Structure of the tail sheath in the extended and contracted conformation

#### Crystal structure of gp18

**Figure 11:**
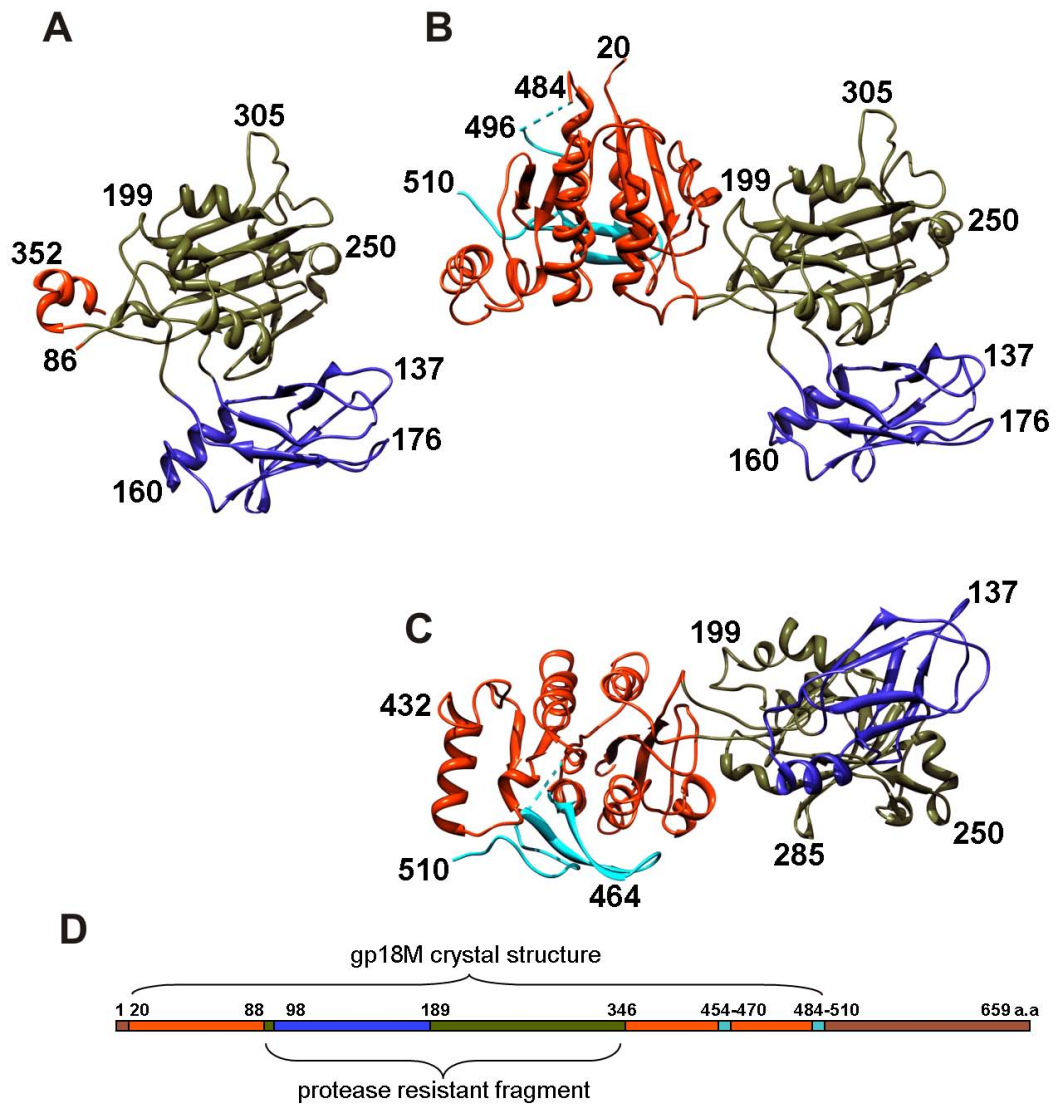
**Structures of the gp18 deletion mutants reprinted from **[53]. **A**, A ribbon diagram of the gp18PR mutant. The N terminus is shown in blue, the C terminus in red and the intermediate residues change color in spectral order. **B, C**, A ribbon diagram of the gp18M mutant (¾ of the total protein length). The three domains are shown in blue (domain I), olive green (domain II) and orange red (domain III); the β-hairpin (residues 454-470) and the last 14 C-terminal residues of gp18M are shown in cyan. **D**, Domain positions on the amino acid sequence, using the same color scheme as in (**B**) and (**C**). Brown indicates the part of gp18 for which the structure remains unknown.

Recombinant, full-length gp18 (659 residues) assembles into tubular polymers of variable lengths called polysheaths, which makes crystallization and high resolution cryoEM studies difficult. However several deletion mutants that lack polymerization properties have been crystallized [52]. The crystal structures of two of these mutants have been determined. One of these is of a protease resistant fragment (gp18PR) consisting of residues 83-365. The other, called gp18M, is of residues 1-510 in which the C-terminal residue has been replaced by a proline (Figure 11). The crystal structure of the gp18PR fragment has been refined to 1.8 Å resolution and the structure of the larger gp18M fragment was determined to 3.5 Å resolution [53].

The structure of gp18M includes that of gp18PR and consists of Domains I, II and III (Figure 11). Domain I (residues 98-188) is a six-stranded β-barrel plus an α-helix. Domain II (residues 88-97 and 189-345) is a two layer β-sandwich, flanked by four small α-helices. Together, domains I and II form the protease resistant fragment gp18PR. Domain III (residues 24-87 and 346-510) consists of a β-sheet with five parallel and one anti-parallel β-strands plus six α-helices surrounding the β-sheet. The 24 N-terminal residues as well as residues 481 to 496 were not ordered in the gp18M crystal structure. The N and C termini of the structure are close in space, suggesting that the first 24 residues and residues 510-659 form an additional domain, Domain IV, which completes the structure of the full-length protein. The overall topology of the gp18 polypeptide chain is quite remarkable. Domain I of gp18 is an insertion into Domain II, which, in turn, is inserted into Domain III, which is inserted between the N and C termini comprising domain IV.

**Figure 12:**
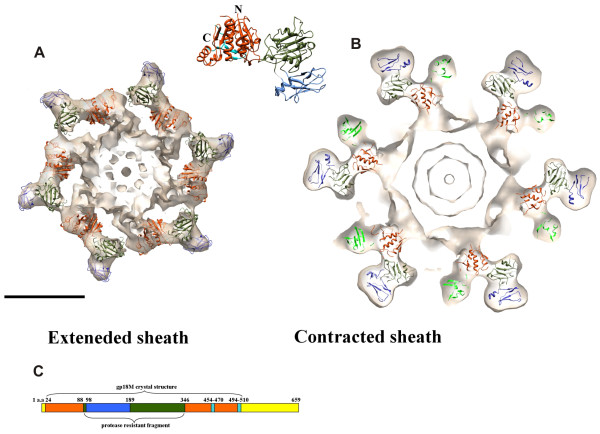
**Arrangement of the gp18 domains in the extended (**A**) and the contracted (**B**) tail reprinted from **[53]. Domains I, II and III of gp18M are colored blue, olive green and orange red, respectively. The same color scheme is used in (**C**) the linear sequence diagram of the full-length gp18 and on the ribbon diagram of the gp18M structure. In (**B**) a part of the domain II from the next disk that becomes inserted between the subunits is shown in bright green. In both extended and contracted sheaths the additional density corresponds to domain IV of gp18 and the tail tube.

Fitting of the gp18M structure into the cryoEM map of the tail showed that the protease resistant part of gp18 is exposed to the solution, whereas the N and C termini, which form Domain IV, are positioned on the interior of the tail sheath (Figure 12). The exposed and buried residues in each conformation of the sheath are in agreement with previous immuno-labeling and chemical modification studies [54,55]. Domain I of gp18 is protruding outwards from the tail and is not involved in inter-subunit contacts. The other three domains form the core of the tail sheath with Domains III and IV being the most conserved parts of tail sheath proteins among T4-related bacteriophages (Figure 12). Despite the fact that Domain I has apparently no role in gp18-gp18 interactions, this domain binds to the baseplate in the extended tail sheath. Thus, one of the roles of Domain I may be to initiate sheath assembly and contraction. Domain I also binds the long tail fibers when they are retracted. It was previously shown that three mutations in Domain I (G106→S, S175→F, A178→V) inhibit fiber retraction [56]. These mutations map to two loops close to the retracted tail fiber attachment site on the surface of the extended tail sheath, presumably abrogating binding of the tail fibers.

### Structure of the extended sheath and the tube

**Figure 13:**
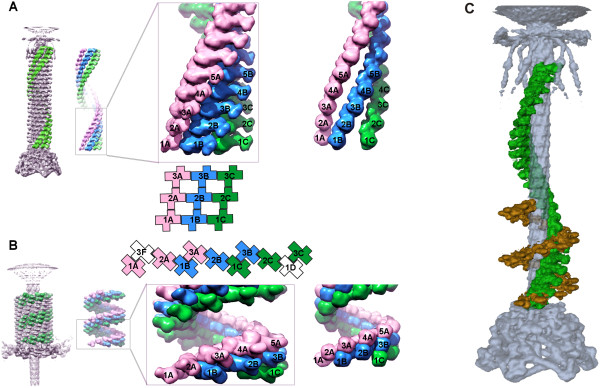
**Connectivity of the sheath subunits in the extended (**A**) and contracted (**B**) tail sheath reprinted from **[53]. The cryoEM map of the entire tail is shown on the far left. Immediately next to it, the three adjacent helices (in pink, blue and green) are shown to permit a better view of the internal arrangement. The successive hexameric discs are numbered 1, 2, 3, 4 and 5 with disc number 1 being closest to the baseplate. In the middle panels are the three helices formed by domains I, II and III. On the right is the arrangement of domain IV, for which the crystal structure is unknown. This domain retains the connectivity between neighboring subunits within each helix in both conformations of the sheath. **C**, One sixth of the gp18 helix - one strand - is shown for the extended (green) and contracted (golden brown) sheath conformations.

**Figure 14:**
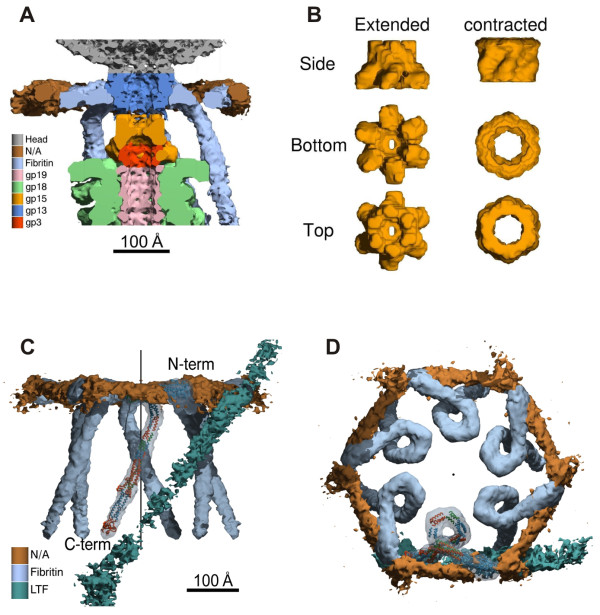
**The structure of the collar and whiskers; reprinted from **[5]. **A**, Cutaway view of the tail neck region. **B**, The structure of the gp15 hexameric ring in the extended and contracted tail. **C**, and **D**, Side and top views of the collar structure. For clarity, only one long tail fiber (LTF) is shown. The uninterpreted density between the fibritin molecules is indicated with brown color and labeled "NA".

The 240 Å-diameter and 925 Å-long sheath is assembled onto the baseplate and terminates with an elaborate 'neck' structure at the other end (Figures 13 and 14). The 138 copies of the sheath protein, gp18, form 23 rings of six subunits each stacked onto one another. Each ring is 40.6 Å thick and is rotated by 17.2° in a right-handed manner relative to the previous ring. The sheath surrounds the tail tube, which has external and internal diameters of 90 Å and 40 Å, respectively. The area of contact between the adjacent gp18 subunits with the neighboring gp18 subunit in the ring above is significantly greater than that between neighboring subunits within a ring (about 2,000 Å^2 ^versus 400 Å^2^). Thus, the sheath is a six-fold-symmetric, six-start helix (Figure 13).

The tail tube (also called the "core" in the literature) is a smooth cylinder, lacking easily discernable surface features. Nevertheless, it can be segmented into individual subunits of the tail tube protein gp19 at an elevated contour level. The subunits are arranged into a helix having the same helical parameters as those found for the gp18 helix.

### Structure of the contracted sheath

The contracted sheath has a diameter of 330 Å and is 420 Å long (Figures 7 and 13). The gp18 subunits form a six-start right-handed helix with a pitch of 16.4 Å and a twist angle of 32.9° situated between radii of 60 Å and 165 Å. The sheath has an inner diameter of 120 Å and does not interact with the 90 Å-diameter tail tube, in agreement with previous observations [57]. Upon superimposing the midsection of the sheath onto itself using the helical transformation, the correlation coefficient was found to be 0.98, showing that there is little variation in the structure of the gp18 subunits and that the sheath contracts uniformly.

The structure of gp18 subunit in the contracted tail is very similar to that in the extended tail. The internal part of the gp18 subunits retains its initial six-start helical connectivity, which is formed when the sheath is first assembled onto the tail tube. This helix has a smaller diameter in the extended conformation and interacts with the tail tube, thus stabilizing the sheath. This was further confirmed by fitting of the gp18M crystal structure into the cryoEM density maps of the tail sheath. The structure fits as a rigid body into both the extended and contracted conformations of the sheath, suggesting that contraction occurs by sliding of individual gp18 subunits over each other with minimal changes to the overall fold of the sheath protein (Figure 12). During contraction each subunit of gp18 moves outwards from the tail axis while slightly changing its orientation. The interactions between the C-terminal domains of gp18 subunits in the extended confirmation appear to be preserved in the contracted form, maintaining the integrity of the sheath structure. However, the outer domains of gp18 change interaction partners and form new contacts. As a result, the interaction area between the subunits increases about four times.

The helical symmetry of the sheath shows that the first and last layers in the extended and contracted conformations are related by a 378.4° (1.05 turns) rotation and 723.8° (2.01 turns) rotation, respectively. Assuming that the association of the sheath and tail tube subunits in the neck region is fixed, the tube will thus rotate by 345.4° - almost a full turn - upon tail contraction (Figure 13C).

Although the diameter of the tube is the same, the symmetry and gp19 subunit organization bear no resemblance to that of the extended or contracted sheath. The tail tube subunits in phage with a contracted tail appear to have an organization that is slightly different to that found in the virus with an extended sheath.. However this might be an artifact of the image reconstruction procedure used to view the details of the tail tube, because the tail tube is internal to the sheath, which has a repetitive structure that might have influenced the reconstruction procedure.

The neck region lacks the fibritin and other proteins in the contracted tail map. This sample was prepared by diluting a concentrated phage specimen into 3 M urea. There is little doubt now, that this harsh treatment caused the observed artifacts. Recent experiments showed that the fibritin and other proteins remain associated with the phage particle if the latter is subjected to slow dialysis into 3 M urea. In this procedure, the tails uniformly contract and their structure is identical to that found in the earlier studies (A. Aksyuk, unpublished observations).

### Structure of the neck region

The neck consists of a several sets of stacked hexameric rings consisting of gp3, gp15, and gp13 or gp14 (Figure 14). The gp3 terminates the tail tube, followed by gp15, and then by gp13 and/or gp14 closest to the head. In the cryoEM reconstruction of the wild type phage, the channel running through the length of the gp19 tube is filled with a roughly continuous density at an average diameter of ~20 Å. This might be the extended molecule(s) of gp29 tape measure protein or phage DNA. The former proposition is more likely, as the tail channel is blocked by the gp15 hexamer, which forms a closed iris with an opening of only 5-10 Å and should prevent the DNA from entering the tail.

The neck is surrounded by a 300 Å diameter and 40 Å thick collar, consisting at least in part of fibritin (gp *wac*) [58]. Fibritin is a 530 Å-long and 20 Å-diameter trimeric fiber [59]. The atomic structure of the N- and C-terminal fragments of fibritin is known [60,61]. The rest of this fiber has a segmented coiled coil structure and can be modeled using the known structure and the repetitive nature of its amino acid sequence [59-61]. The cryoEM map of wild type T4 could be interpreted with the help of this model.

Each of the six fibritin trimers forms a tight 360° loop, which together create the main part of the collar and the whiskers (Figure 14). Both the N and C termini of the fibritin protein attach to the long tail fiber. The C-terminal end binds to the 'kneecap' region of the long tail fiber, comprised of gp35, whereas the N terminus most probably binds to the junction region of gp36 and gp37. The fibritin's 360° loop interacts with gp15 and is in the N-terminal part of the protein. This is in agreement with earlier studies that found that the N terminus of fibritin is required for its attachment to the phage particle. The six fibritins and the long tail fibers are bridged together by six copies of an unknown fibrous protein to form a closed ring. This protein is about 160 Å long and 35 Å in diameter.

## Tail Fiber Structure and Assembly

### Overall organization and subunit composition

The long tail fibers of bacteriophage T4 are kinked structures of about 1440 Å long with a variable width of up to about 50 Å. They can be divided into proximal and distal half-fibers, attached at an angle of about 20° [62]. In adverse conditions for phage multiplication, the long tail fibers are in a retracted conformation, lying against the tail sheath and head of the bacteriophage. In the extended conformation, only the proximal end of the fiber is attached to the baseplate. The long tail fibers are responsible for initial interaction with receptor molecules [2]. The distal tip of the long tail fibers can recognize the outer membrane protein C (ompC) or the glucosyl-α-1,3-glucose terminus of rough LPS on *E. coli *[63]. Titration experiments showed that the phage particle has to carry at least three long tail fibers to be infectious [64].

**Figure 15:**
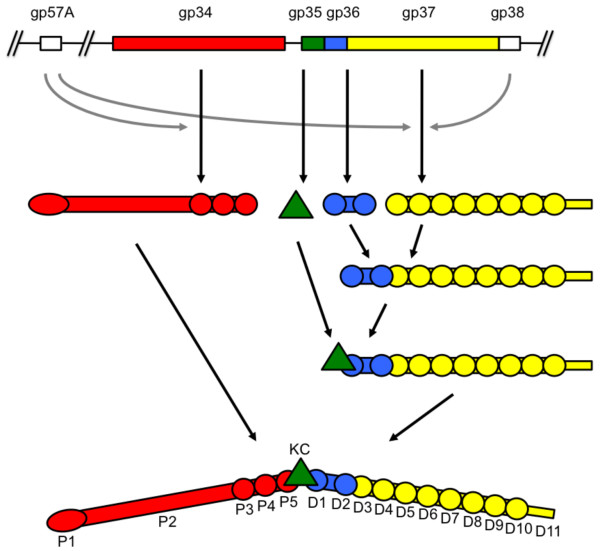
**Gene structure, assembly pathway and domain organization of the bacteriophage T4 long tail fibers**. Chaperone interactions are shown as grey arrows. Domains of the proximal tail fiber are named P1-5 and of the distal half D1-11; gp35, or the knee-cap (KC) is represented as a green triangle.

The long tail fiber is composed of four different gene products: gp34, gp35, gp36 and gp37 (Figure 15) [65]. The proximal half-fiber, or the "thigh", is formed by a parallel homo-trimer of gp34 (1289 amino acids or 140 kDa per monomer). In the intact phage, the N-terminal end of gp34 is attached to the baseplate protein gp9 [8], while the C-terminal end interacts with the distal half-fiber, presumably with gp35 and/or gp36. Gp35 (372 residues; 40 kDa and present as a monomer) forms the "knee" and may be responsible for the angle between the proximal and distal half-fibers. The distal half-fiber is composed of gp35, trimeric gp36 (221 amino acids, 23 kDa) and gp37 (1026 amino acids; 109 kDa). The gp36 protein subunit is located at the proximal end of the distal half-fiber, forming the upper part of the "shin", while gp37 makes up the rest of the shin, including the very distal receptor-recognizing tip (or "foot"), which corresponds to the C-terminal region of gp37.

The four structural genes of the long tail fiber and the chaperone gp38 are located together in the T4 genome. Genes 34 and 35 are co-transcribed from a middle-mode promoter, gene 36 from a late promoter, while genes 37 and 38 are co-transcribed from another promoter [66]. The gp34 protein is the largest T4 protein, followed by the baseplate protein gp7 the second-largest protein and gp37 the third-largest protein in the baseplate.

Despite their extended dimensions, the long tail fibers appear to be stiff structures, because no kinked half-fibers have been observed in electron micrographs. Moreover, the angle between the half fibers in the complete fiber does not deviate very far from 20° on average. The stiffness may be necessary for transmitting the receptor recognition signal from the tip of the fiber to the baseplate and for bringing the phage particle closer to the cell surface as the baseplate changes its conformation. No atomic resolution structures for the long tail fibers, their components or their chaperones (see next section) have yet been published.

In the cryoEM reconstruction of the wild-type T4, the fibers are in the retracted configuration (Figure 7), likely caused by the unfavorable for infection conditions of the cryoEM imaging procedure (a very high phage concentration and a very low salt buffer). The density corresponding to the long tail fibers is quite poor (Figure 7). This is likely caused by the variability of the positions of the long tail fibers. The 700 Å-long proximal half-fiber and the about 2/3 of the 740 Å-long distal part are present in the cryoEM map. The proximal half-fiber is bent around the sheath, forming about a quarter of a right-handed helix.

### Assembly: folding chaperones and attachment proteins

A phage-encoded molecular chaperone, gp57A, is required for the correct trimerization of long tail fiber proteins gp34 and gp37 [62]; and for the short tail fiber protein gp12 [67] (Table 2). Gp57A appears to be a rather general T4 tail fiber chaperone and is needed for the correct assembly of the trimeric short and long tail fiber proteins gp12, gp34, and gp37 [68]. Gp57A is a small protein of 79 residues (8,613 Da) that lacks aromatic amino acids, cysteines and prolines. *In vitro*, it adopts different oligomeric states [44]. The specific chaperone gp38 must be present [68] for the correct trimeric assembly of gp37. The molecular basis of the gp38 and gp57A chaperone activities are unclear, but it has been proposed that gp57A functions to keep fiber protein monomers from aggregating unspecifically, while gp38 may bring together the C-terminal ends of the monomers to start the folding process [62]. Qu *et al. *[69] noted that extension of a putative coiled-coil motif near the C-terminal end of gp37 bypasses the need for the gp38 chaperone. The extended coiled-coil may function as an intramolecular clamp, obviating the need for the intermolecular gp38 chaperone.

Two parts of the long tail fiber (the distal and proximal half-fibers) assemble independently. The three proteins of the distal half-fiber interact in the following order. Initially trimeric gp36 binds to the N-terminal region of gp37, and then monomeric gp35 binds to gp36, completing the assembly of the distal half-fiber. Joining of the two half-fibers presumably takes place spontaneously.

Attachment of the assembled long tail fiber to the phage particle is promoted by gp63 and the fibritin (gp *wac*) [62], although neither of these proteins is absolutely essential (Table 2). Unlike gp63, the fibritin is a component of the complete phage particle and constitutes a major part of the neck complex (see above). In the absence of the fibritin, the long tail fibers attach to fiberless particles very slowly. The whiskers are also be involved in the retraction of the long tail fibers under unfavorable conditions. Gp63 has RNA ligase activity and may function as such in infected cells. However, the isolation of gene 63 mutants that affect RNA ligase activity, but not tail fiber attachment activity suggests that gp63 is a bifunctional protein that promotes two physiologically unrelated reaction [70].

### Structural studies of the long tail fiber

Scanning transmission electron microscopy of stained and unstained particles has been used to study the structure of intact long tail fibers, proximal half-fibers and distal half-fibers [65]. The proximal half-fiber, gp34, consists of an N-terminal globular domain that interacts with the baseplate. It is followed by a rod-like shaft about 400 Å long that is connected to the globular domain by a hinge. The rod domain seen by EM correlates with a cluster of seven quasi-repeats (residues 438 to 797 [65]), which are also present six times in gp12 and once in gp37. One of these repeats is resolved in the crystal structure of gp12 (amino acids 246 to 290 [12]). This structural motif consists of an α-helix and a β-sheet. The proximal half-fiber terminates in three globular domains arranged like beads on a stick.

EM has shown that the proximal and distal half-fibers are connected at an angle of about 160°. A hinge is present between the proximal and distal half-fibers, forming the "knee". Density, associated with the presence of gp35, a monomer in the long tail fiber, bulges asymmetrically out on the side of the fiber forming the reflex angle (i.e. at the opposite side of the obtuse angle) [65].

The distal half-fiber, composed of gp36 and gp37, consists of ten globular domains of variable size and spacing, preceding a thin end domain or "needle" with dimensions of about 150 by 25 Å [65]. Based on its relative molecular mass (compared to that of the other long tail fiber components), gp36 should make up about one sixth of the distal half-fiber and thus likely composes at least the two relatively small proximal globules, the thin rod in between them, and perhaps the third globule. The remaining seven or eight globules and the needle or "foot" would then be gp37. A single repeat, similar to those also present in gp12 and gp34 is found in the N-terminal region of gp37, (amino acids 88-104). Residues 486 to 513 of gp37 show strong similarity to residues 971 to 998 of gp34 and are likely to form a homologous structural motif. Another sequence similarity has been observed between residues 814-860 and residues 342-397 of gp12 [65]. In gp12, these residues form the collar domain [12,14]. Gp34, gp36, and gp37 are predicted to mainly contain β-structure and little α-helical structure. However, their limited sequence similarity with each other, with the T4 short tail fiber protein gp12 and with other fiber proteins makes structure prediction difficult. *Streptococcus pyogenes *prophage tail fiber was shown to contain an extended triple β-helix in between α-helical triple coiled-coil regions [71], while the bacteriophage P22 tail needle gp26 has a very small triple β-helical domain and extensive stable α-helical triple coiled-coil regions [72]. A general principle may be that folding of the above mentioned fiber proteins starts near the C-terminus, as is the case for the adenovirus vertex fibers [73].

In general, trimeric fibrous proteins require a chaperone 'module' for folding. This module can be a small domain of the same polypeptide chain or a separate protein (or several proteins) [74]. Simultaneous co-expression of gp37, gp57A and gp38 has been used to obtain mg-amounts of soluble gp37 [75]. Correct folding of the trimeric protein was assessed by gel electrophoresis, cross-linking and transmission electron microscopy studies. The C-terminal fragments of gp37 appear to be folded correctly, showing that folding behavior of gp37 resembles that of gp12 [38].

## The Infection Mechanism

### Structural transformation of the tail during infection

The following observations suggest that the hexagonal conformation of the baseplate and the extended state of the sheath both represent high energy metastable assemblies. Purified baseplates have been shown to switch spontaneously into the star conformation [50]. In the absence of either the baseplate or the tail tube, the sheath assembles into a long tubular structure similar to that of the contracted sheath [57]. The tail sheath contraction is irreversible, and the contracted tail structure is resistant to 8 M urea [76]. These observations suggest that the baseplate in the hexagonal conformation together with its extended sheath can be compared to an extended spring ready to be triggered [77].

**Figure 16:**
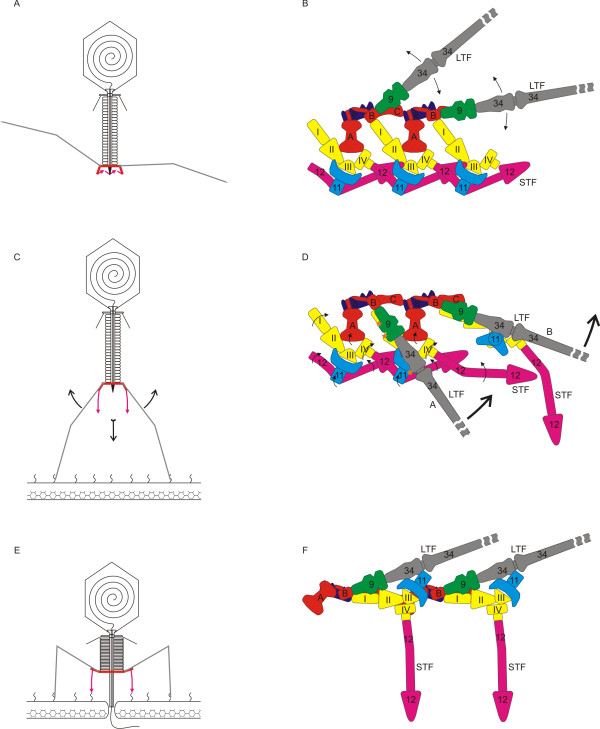
**Baseplate conformational switch schematic reprinted from **[6]. **A **and **B**, The phage is free in solution. The long tail fibers are extended and oscillate around their midpoint position. The movements of the fibers are indicated with black arrows. The proteins are labeled with their corresponding gene numbers and colored as in other figures. **C **and **D**, The long tail fibers attach to their surface receptors and adapt the "down" conformation. The fiber labeled "A" and its corresponding attachment protein gp9 interact with gp11 and with gp10, respectively. These interactions, labeled with orange stars, probably initiate the conformational switch of the baseplate. The black arrows indicate tentative domain movements and rotations, which have been derived from the comparison of the two terminal conformations. The fiber labeled "B" has advanced along the conformational switch pathway so that gp11 is now seen along its threefold axis and the short tail fiber is partially extended in preparation for binding to its receptor. The thick red arrows indicate the projected movements of the fibers and the baseplate. **E **and **F**, The conformational switch is complete; the short tail fibers have bound their receptors and the sheath has contracted. The phage has initiated DNA transfer into the cell.

By combining all the available experimental information on T4 infection, it is possible to describe the process of attachment of the phage to the host cell in some detail (Figure 16**, Movie 2 **http://www.seyet.com/t4_virology.html). The long tail fibers of the infectious phage in solution are extended, and most possibly move up and down due to the thermal motion [51,78,79]. Attachment of one of the fibers to the cell surface increases the probability for the other fibers to find cell surface receptors. The attachment of three or more of the long tail fibers to their host cell receptors is possible only when they point towards the host cell surface. This configuration of the tail fibers orients the phage particle perpendicular to the cell surface.

As the gp9 trimer is coaxial with the proximal part of the long tail fiber, gp9 proteins swivel up and down following the movements of the long tail fibers as the phage particle travels in search of a potential host cell. When the long tail fibers attach to the host cell surface and their proximal parts point down, several new protein-protein interactions at the periphery of the baseplate are initiated: 1) gp9 binds to the C-terminal domain of gp10; 2) the long tail fiber binds to a gp11 trimer. These interactions are likely causing gp11 to dissociate from gp12 leading to destabilization of the gp12 garland. The baseplate then unlocks from its high energy metastable hexagonal state. The A domain of gp7 swivels outwards and the entire gp10-gp11-gp12 module rotates, causing the C-terminal domains of the short tail fibers to point towards the host cell surface, thus preparing them for binding to the host cell receptors. Gp9 and the long tail fibers remain bound to the baseplate pins (the gp7-gp10-gp11 module), during this transformation.

During the conformational change of the baseplate, the long tail fibers are being used as levers to move the baseplate towards the cell surface by as much as 1000 Å. As the lengths of the two halves of the fiber are close to 700 Å each, such a large translation is accomplished by changing the angle between them by about 100°.

The conformational changes, which are initiated at the periphery of the baseplate, would then spread inwards into the center of the baseplate causing the central part of the baseplate (gp6, gp25 and gp53) to alter its conformation and thus initiating sheath contraction. The process of sheath contraction is accomplished by rotating and sliding the gp18 sheath subunits and progresses through the entire sheath starting at the baseplate (**Movie 3 **http://www.seyet.com/t4_virology.html). The contracting sheath then drives the tail tube into the host membrane. The baseplate hub, which is positioned at the tip of the tube, will be the first to come in contact with the membrane. The membrane is then punctured with the help of the gp5 C-terminal β-helix and the yet unidentified protein (gp28?), which caps the tip of the gp5 β-helix. Subsequent tail contraction drives the tail tube further, and the entire gp5-gp27 complex is then translocated into the periplasmic space. The three lysozyme domains of the gp5 trimer start their digestion of peptidoglycan after the gp5 β-helix has dissociated due to the steric clashes with the peptidoglycan. This process results is a hole in the outer part of the cell envelope, allowing the tail tube to interact with the cytoplasmic membrane initiating phage DNA transfer. As mentioned above, the tail contraction involves rotation of the tail tube by an almost complete turn. Thus, the tail tube drills, rather than punctures, the outer membrane.

The fate and function of gp27 in the infection is unknown. Gp27 does not appear to form a trimer in the absence of gp5 [13], but it is possible that gp27 might be able to maintain its trimeric form upon its association with the tail tube because the gp27 trimer is a smooth coaxial continuation of the tail tube with a 25 Å diameter channel. Furthermore, the lysozyme-containing N-terminal part of gp5 (gp5*) might be able to dissociate from gp27 in the periplasm (due to the lower pH [13]) to open the gp27 channel. Gp27 may thus form the last terminal pore of the tube through which the phage DNA and proteins enter the host cell. Possibly, gp27 might interact with a receptor in or at the cytoplasmic membrane.

The above speculation that the gp27 trimer may serve as the terminal opening of the tail tube is supported by the crystal structure of a gp27 homolog called gp44 from bacteriophage Mu (a contractile tail phage) [80]. Although T4 gp27 and Mu gp44 have no detectible sequence similarity, the two structures have very similar folds [80]. Gp44, however, forms a stable trimer in solution and most probably serves as a centerpiece of the Mu baseplate. Gp45 is a glycine-rich protein from the Mu tail, making it a possible ortholog of gp5.

## Conclusion

### Contractile tail evolution and relation to other biological systems

There is building a body of evidence proving that all tailed phages have a common ancestor. The evolutionary relationship cannot be detected in their amino acid sequences, but structural studies show that capsid proteins of all tailed phages have a common fold (the HK97 fold) and that the portal proteins are homologous [81-83]. As the DNA packaging processes in all tailed phages are similar, their ATPases and many other structural proteins are also most probably homologous.

The recently discovered and incompletely characterized bacterial type VI secretion system (T6SS) appears to be related to a phage tail [84]. The T6SS is one of the most common secretion systems present in at least 25% of all Gram-negative bacteria, and is associated with an increased virulence of many pathogens [85]. Similar to other secretion systems, T6SS genes are clustered in pathogenicity islands containing 20 or more open reading frames. The hallmark of the T6SS expression is the presence of the conserved Hcp protein in the external medium [86]. VgrG proteins represent the other most common type of protein found secreted in a T6SS-dependent fashion. It was shown that in *Vibrio cholerae*, VgrG-1 is responsible for T6SS-dependent cytotoxic effects of *V. cholerae *on host cells including *Dictyostelium discoideum *amoebae and J774 macrophages [87]. The C terminus of VgrG-1 encodes a 548 residue-long actin cross-linking domain or ACD [87], which is also found embedded in a secreted toxin of *V. cholerae *called RtxA. VgrG orthologs in bacterial species other than *V. cholerae *carry a wide range of putative effector domains fused to their C termini [87].

The crystal structure of the N-terminal fragment the *Escherichia coli *CFT073 VgrG protein encoded by ORF c3393 shows a significant structural similarity to the gp5-gp27 complex, despite only 13% sequence identity [84]. The crystal structure of Hcp1 [88], the most abundant secreted protein in T6SS-expressing *Pseudomonas aeruginosa *strain PAO1, shows that it is homologous to the tandem 'tube' domain of gp27, which interacts with the T4 tail tube. Hcp1 is a donut-shaped hexamer with external and internal diameters of 85 Å and 40 Å, respectively. These hexamers stack on top of each other head-to-tail to form continuous tubes in the crystals. Some Hcp proteins can form tubes *in vitro *[84]. The homology of these two key proteins to the phage tail proteins and the fact that VgrG is translocated across a lipid membrane into a target cell suggest that the T6SS machine and phage tails might have a common ancestor.

Many evolutionary questions deal with the chicken and egg paradox. Whether the phage tail has evolved from the T6SS or vice versa is one of those questions. Clearly, the phage and its host benefit from coexistence and are capable of exchanging not only small proteins and protein domains, but also large and sophisticated supramolecular assemblies.

## Abbreviations

Gp: gene product; HEWL: hen egg white lysozyme; LPS: lipopolysaccharide; NAG: N-acetylglucosamine; NAM: N-acetlymuramic acid; ORF: open reading frame; RMSD: root mean square deviation; T4L: lysozyme of T4 phage encoded by gene *e*; T6SS: bacterial type VI secretion system.

## Competing interests

The authors declare that they have no competing interests.

## Authors' contributions

PGL, FA and MJvR made a summary of the available data and wrote the text. MGR carried out a major revision of the text. PGL, VAK, AAA and SK prepared the figures. PGL and Seyet LLC created Movies 1 and 2. AAA created Movie 3. All authors read and approved the final manuscript.

## References

[B1] Ackermann HW Bacteriophage observations and evolution Res Microbiol 2003 154 245 251 10.1016/S0923-2508(03)00067-6 12798228

[B2] Goldberg E Grinius L Letellier L Karam JD Recognition, attachment, and injection Molecular Biology of Bacteriophage T4 1994 Washington, D.C.: American Society for Microbiology 347 356

[B3] Epstein RH Bolle A Steinberg C Kellenberger E Boy de la Tour E Chevalley R Edgar R Susman M Denghardt C Lielausis I Physiological studies of conditional lethal mutants of bacteriophage T4D Cold Spring Harbor Symposia on Quantitative Biology 1963 28 375 392

[B4] Eiserling FA Black LW Karam JD Pathways in T4 morphogenesis Molecular Biology of Bacteriophage T4 1994 Washington, D.C.: American Society for Microbiology 209 212

[B5] Kostyuchenko VA Leiman PG Chipman PR Kanamaru S van Raaij MJ Arisaka F Mesyanzhinov VV Rossmann MG Three-dimensional structure of bacteriophage T4 baseplate Nat Struct Biol 2003 10 688 693 10.1038/nsb970 12923574

[B6] Leiman PG Chipman PR Kostyuchenko VA Mesyanzhinov VV Rossmann MG Three-dimensional rearrangement of proteins in the tail of bacteriophage T4 on infection of its host Cell 2004 118 419 429 10.1016/j.cell.2004.07.022 15315755

[B7] Aksyuk AA Leiman PG Shneider MM Mesyanzhinov VV Rossmann MG The structure of gene product 6 of bacteriophage T4, the hinge-pin of the baseplate Structure 2009 17 800 808 10.1016/j.str.2009.04.005 19523898

[B8] Kostyuchenko VA Navruzbekov GA Kurochkina LP Strelkov SV Mesyanzhinov VV Rossmann MG The structure of bacteriophage T4 gene product 9: the trigger for tail contraction Structure Fold Des 1999 7 1213 1222 10.1016/S0969-2126(00)80055-6 10545330

[B9] Leiman PG Kostyuchenko VA Shneider MM Kurochkina LP Mesyanzhinov VV Rossmann MG Structure of bacteriophage T4 gene product 11, the interface between the baseplate and short tail fibers J Mol Biol 2000 301 975 985 10.1006/jmbi.2000.3989 10966799

[B10] Leiman PG Shneider MM Kostyuchenko VA Chipman PR Mesyanzhinov VV Rossmann MG Structure and location of gene product 8 in the bacteriophage T4 baseplate J Mol Biol 2003 328 821 833 10.1016/S0022-2836(03)00366-8 12729757

[B11] Leiman PG Shneider MM Mesyanzhinov VV Rossmann MG Evolution of bacteriophage tails: Structure of T4 gene product 10 J Mol Biol 2006 358 912 921 10.1016/j.jmb.2006.02.058 16554069

[B12] van Raaij MJ Schoehn G Burda MR Miller S Crystal structure of a heat and protease-stable part of the bacteriophage T4 short tail fibre J Mol Biol 2001 314 1137 1146 10.1006/jmbi.2000.5204 11743729

[B13] Kanamaru S Leiman PG Kostyuchenko VA Chipman PR Mesyanzhinov VV Arisaka F Rossmann MG Structure of the cell-puncturing device of bacteriophage T4 Nature 2002 415 553 557 10.1038/415553a 11823865

[B14] Thomassen E Gielen G Schutz M Schoehn G Abrahams JP Miller S van Raaij MJ The structure of the receptor-binding domain of the bacteriophage T4 short tail fibre reveals a knitted trimeric metal-binding fold J Mol Biol 2003 331 361 373 10.1016/S0022-2836(03)00755-1 12888344

[B15] Rossmann MG Arisaka F Battisti AJ Bowman VD Chipman PR Fokine A Hafenstein S Kanamaru S Kostyuchenko VA Mesyanzhinov VV Shneider MM Morais MC Leiman PG Palermo LM Parrish CR Xiao C From structure of the complex to understanding of the biology Acta Crystallogr D Biol Crystallogr 2007 63 9 16 10.1107/S0907444906047330 17164521 PMC2483488

[B16] Berget PB King J Isolation and characterization of precursors in T4 baseplate assembly. The complex of gene 10 and gene 11 products J Mol Biol 1978 124 469 486 10.1016/0022-2836(78)90182-1 712843

[B17] Coombs DH Arisaka F Karam JD T4 tail structure and function Molecular Biology of Bacteriophage T4 1994 Washington, D.C.: American Society for Microbiology 259 281

[B18] Leiman PG Kanamaru S Mesyanzhinov VV Arisaka F Rossmann MG Structure and morphogenesis of bacteriophage T4 Cell Mol Life Sci 2003 60 2356 2370 10.1007/s00018-003-3072-1 14625682 PMC11138918

[B19] Mosig G Eiserling F R C, ST A T4 and related phages: structure and development The Bacteriophages 2006 Oxford: Oxford University Press

[B20] Kikuchi Y King J Assembly of the tail of bacteriophage T4 J Supramol Struct 1975 3 24 38 10.1002/jss.400030104 1152465

[B21] Kikuchi Y King J Genetic control of bacteriophage T4 baseplate morphogenesis. III. Formation of the central plug and overall assembly pathway J Mol Biol 1975 99 695 716 10.1016/S0022-2836(75)80180-X 765483

[B22] Kikuchi Y King J Genetic control of bacteriophage T4 baseplate morphogenesis. II. Mutants unable to form the central part of the baseplate J Mol Biol 1975 99 673 694 10.1016/S0022-2836(75)80179-3 765482

[B23] Kikuchi Y King J Genetic control of bacteriophage T4 baseplate morphogenesis. I. Sequential assembly of the major precursor, in vivo and in vitro J Mol Biol 1975 99 645 672 10.1016/S0022-2836(75)80178-1 765481

[B24] Ferguson PL Coombs DH Pulse-chase analysis of the in vivo assembly of the bacteriophage T4 tail J Mol Biol 2000 297 99 117 10.1006/jmbi.2000.3551 10704310

[B25] Yap ML Mio K Leiman PG Kanamaru S Arisaka F The baseplate wedges of bacteriophage T4 spontaneously assemble into hubless baseplate-like structure in vitro J Mol Biol 2010 395 349 360 10.1016/j.jmb.2009.10.071 19896486

[B26] Snustad DP Dominance interactions in Escherichia coli cells mixedly infected with bacteriophage T4D wild-type and amber mutants and their possible implications as to type of gene-product function: catalytic vs. stoichiometric Virology 1968 35 550 563 10.1016/0042-6822(68)90285-7 4878023

[B27] Nieradko J Koszalka P Evidence of interactions between Gp27 and Gp28 constituents of the central part of bacteriophage T4 baseplate Acta Microbiol Pol 1999 48 233 242 10756710

[B28] Abuladze NK Gingery M Tsai J Eiserling FA Tail length determination in bacteriophage T4 Virology 1994 199 301 310 10.1006/viro.1994.1128 8122363

[B29] Akhter T Zhao L Kohda A Mio K Kanamaru S Arisaka F The neck of bacteriophage T4 is a ring-like structure formed by a hetero-oligomer of gp13 and gp14 Biochim Biophys Acta 2007 1774 1036 1043 17602902 10.1016/j.bbapap.2007.05.011

[B30] Kao SH McClain WH Baseplate protein of bacteriophage T4 with both structural and lytic functions J Virol 1980 34 95 103 6990017 10.1128/jvi.34.1.95-103.1980 PMC288674

[B31] Mosig G Lin GW Franklin J Fan WH Functional relationships and structural determinants of two bacteriophage T4 lysozymes: a soluble (gene e) and a baseplate-associated (gene 5) protein New Biol 1989 1 171 179 2488704

[B32] Matthews BW Remington SJ The three dimensional structure of the lysozyme from bacteriophage T4 Proc Natl Acad Sci USA 1974 71 4178 4182 10.1073/pnas.71.10.4178 4530293 PMC434353

[B33] Kanamaru S Gassner NC Ye N Takeda S Arisaka F The C-terminal fragment of the precursor tail lysozyme of bacteriophage T4 stays as a structural component of the baseplate after cleavage J Bacteriol 1999 181 2739 2744 10217762 10.1128/jb.181.9.2739-2744.1999 PMC93713

[B34] Murzin AG OB(oligonucleotide/oligosaccharide binding)-fold: common structural and functional solution for non-homologous sequences Embo J 1993 12 861 867 8458342 10.1002/j.1460-2075.1993.tb05726.x PMC413284

[B35] Arcus V OB-fold domains: a snapshot of the evolution of sequence, structure and function Curr Opin Struct Biol 2002 12 794 801 10.1016/S0959-440X(02)00392-5 12504685

[B36] Kuroki R Weaver LH Matthews BW A covalent enzyme-substrate intermediate with saccharide distortion in a mutant T4 lysozyme Science 1993 262 2030 2033 10.1126/science.8266098 8266098

[B37] Zorzopulos J Kozloff LM Identification of T4D bacteriophage gene product 12 as the baseplate zinc metalloprotein J Biol Chem 1978 253 5543 5547 353052

[B38] van Raaij MJ Schoehn G Jaquinod M Ashman K Burda MR Miller S Identification and crystallisation of a heat- and protease-stable fragment of the bacteriophage T4 short tail fibre Biol Chem 2001 382 1049 1055 10.1515/BC.2001.131 11530935

[B39] Leiman PG Molineux IJ Evolution of a new enzyme activity from the same motif fold Mol Microbiol 2008 69 287 290 10.1111/j.1365-2958.2008.06241.x 18433454 PMC2574927

[B40] Stummeyer K Dickmanns A Muhlenhoff M Gerardy-Schahn R Ficner R Crystal structure of the polysialic acid-degrading endosialidase of bacteriophage K1F Nat Struct Mol Biol 2005 12 90 96 10.1038/nsmb874 15608653

[B41] Stummeyer K Schwarzer D Claus H Vogel U Gerardy-Schahn R Muhlenhoff M Evolution of bacteriophages infecting encapsulated bacteria: lessons from Escherichia coli K1-specific phages Mol Microbiol 2006 60 1123 1135 10.1111/j.1365-2958.2006.05173.x 16689790

[B42] Spinelli S Desmyter A Verrips CT de Haard HJ Moineau S Cambillau C Lactococcal bacteriophage p2 receptor-binding protein structure suggests a common ancestor gene with bacterial and mammalian viruses Nat Struct Mol Biol 2006 13 85 89 10.1038/nsmb1029 16327804

[B43] Matsui T Griniuviene B Goldberg E Tsugita A Tanaka N Arisaka F Isolation and characterization of a molecular chaperone, gp57A, of bacteriophage T4 J Bacteriol 1997 179 1846 1851 9068627 10.1128/jb.179.6.1846-1851.1997 PMC178905

[B44] Ali SA Iwabuchi N Matsui T Hirota K Kidokoro S Arai M Kuwajima K Schuck P Arisaka F Reversible and fast association equilibria of a molecular chaperone, gp57A, of bacteriophage T4 Biophys J 2003 85 2606 2618 10.1016/S0006-3495(03)74683-9 14507723 PMC1303484

[B45] Haggard-Ljungquist E Jacobsen E Rishovd S Six EW Nilssen O Sunshine MG Lindqvist BH Kim KJ Barreiro V Koonin EV Calendar R Bacteriophage P2: genes involved in baseplate assembly Virology 1995 213 109 121 10.1006/viro.1995.1551 7483254

[B46] Morgan GJ Hatfull GF Casjens S Hendrix RW Bacteriophage Mu genome sequence: analysis and comparison with Mu-like prophages in Haemophilus, Neisseria and Deinococcus J Mol Biol 2002 317 337 359 10.1006/jmbi.2002.5437 11922669

[B47] Kostyuchenko VA Chipman PR Leiman PG Arisaka F Mesyanzhinov VV Rossmann MG The tail structure of bacteriophage T4 and its mechanism of contraction Nat Struct Mol Biol 2005 12 810 813 10.1038/nsmb975 16116440

[B48] Watts NR Coombs DH Analysis of near-neighbor contacts in bacteriophage T4 wedges and hubless baseplates by using a cleavable chemical cross-linker J Virol 1989 63 2427 2436 2724408 10.1128/jvi.63.6.2427-2436.1989 PMC250693

[B49] Watts NR Coombs DH Structure of the bacteriophage T4 baseplate as determined by chemical cross-linking J Virol 1990 64 143 154 2403438 10.1128/jvi.64.1.143-154.1990 PMC249069

[B50] Watts NR Hainfeld J Coombs DH Localization of the proteins gp7, gp8 and gp10 in the bacteriophage T4 baseplate with colloidal gold:F(ab)2 and undecagold:Fab' conjugates J Mol Biol 1990 216 315 325 10.1016/S0022-2836(05)80323-7 2254933

[B51] Kellenberger E Stauffer E Haner M Lustig A Karamata D Mechanism of the long tail-fiber deployment of bacteriophages T-even and its role in adsorption, infection and sedimentation Biophys Chem 1996 59 41 59 10.1016/0301-4622(95)00117-4 8867326

[B52] Efimov VP Kurochkina LP Mesyanzhinov VV Engineering of bacteriophage T4 tail sheath protein Biochemistry (Mosc) 2002 67 1366 1370 10.1023/A:1021857926152 12600265

[B53] Aksyuk AA Leiman PG Kurochkina LP Shneider MM Kostyuchenko VA Mesyanzhinov VV Rossmann MG The tail sheath structure of bacteriophage T4: a molecular machine for infecting bacteria Embo J 2009 28 821 829 10.1038/emboj.2009.36 19229296 PMC2670864

[B54] Arisaka F Takeda S Funane K Nishijima N Ishii S Structural studies of the contractile tail sheath protein of bacteriophage T4. 2. Structural analyses of the tail sheath protein, gp18, by limited proteolysis, immunoblotting, and immunoelectron microscopy Biochemistry 1990 29 5057 5062 10.1021/bi00473a009 2143080

[B55] Takeda S Arisaka F Ishii S Kyogoku Y Structural studies of the contractile tail sheath protein of bacteriophage T4. 1. Conformational change of the tail sheath upon contraction as probed by differential chemical modification Biochemistry 1990 29 5050 5056 10.1021/bi00473a008 2143079

[B56] Takeda Y Suzuki M Yamada T Kageyama F Arisaka F Mapping of functional sites on the primary structure of the contractile tail sheath protein of bacteriophage T4 by mutation analysis Biochim Biophys Acta (BBA) - Proteins & Proteomics 2004 1699 163 171 10.1016/j.bbapap.2004.02.010 15158724

[B57] Amos LA Klug A Three-dimensional image reconstructions of the contractile tail of T4 bacteriophage J Mol Biol 1975 99 51 64 10.1016/S0022-2836(75)80158-6 1206701 PMC3920174

[B58] Dewey MJ Wiberg JS Frankel FR Genetic control of whisker antigen of bacteriophage T4D J Mol Biol 1974 84 625 634 10.1016/0022-2836(74)90120-X 4601386

[B59] Efimov VP Nepluev IV Sobolev BN Zurabishvili TG Schulthess T Lustig A Engel J Haener M Aebi U Venyaminov SY Potekhin SA Mesyanzhinov VV Fibritin encoded by bacteriophage T4 gene wac has a parallel triple-stranded alpha-helical coiled-coil structure J Mol Biol 1994 242 470 486 10.1006/jmbi.1994.1595 7932704

[B60] Boudko SP Londer YY Letarov AV Sernova NV Engel J Mesyanzhinov VV Domain organization, folding and stability of bacteriophage T4 fibritin, a segmented coiled-coil protein Eur J Biochem 2002 269 833 841 10.1046/j.1432-1033.2002.02734.x 11846809

[B61] Tao Y Strelkov SV Mesyanzhinov VV Rossmann MG Structure of bacteriophage T4 fibritin: a segmented coiled coil and the role of the C-terminal domain Structure 1997 5 789 798 10.1016/S0969-2126(97)00233-5 9261070

[B62] Wood WB Eiserling FA Crowther RA Karam JD Long tail fibers: genes, proteins, structure, and assembly Molecular Biology of Bacteriophage T4 1994 Washington, D.C.: American Society for Microbiology 282 290

[B63] Henning U Hashemolhosseini S Karam JD Receptor Recognition by T-Even-Type Coliphages Molecular Biology of Bacteriophage T4 1994 Washiington, D.C.: American Society for Microbiology 291 298

[B64] Wood WB Henninger M Attachment of tail fibers in bacteriophage T4 assembly: some properties of the reaction in vitro and its genetic control J Mol Biol 1969 39 603 618 10.1016/0022-2836(69)90148-X 5357214

[B65] Cerritelli ME Wall JS Simon MN Conway JF Steven AC Stoichiometry and domainal organization of the long tail-fiber of bacteriophage T4: a hinged viral adhesin J Mol Biol 1996 260 767 780 10.1006/jmbi.1996.0436 8709154

[B66] Kutter E Guttman B Batts D Peterson S Djavakhishvili T Stidham T Arisaka F Mesyanzhinov V Ruger W Mosig G O'Brien SJ Genomic map of bacteriophage T4 Genomic Maps 1993 New York: Cold Spring Harbor Laboratory Press 1 27

[B67] Burda MR Miller S Folding of coliphage T4 short tail fiber in vitro. Analysing the role of a bacteriophage-encoded chaperone Eur J Biochem 1999 265 771 778 10.1046/j.1432-1327.1999.00782.x 10504409

[B68] Hashemolhosseini S Stierhof YD Hindennach I Henning U Characterization of the helper proteins for the assembly of tail fibers of coliphages T4 and lambda J Bacteriol 1996 178 6258 6265 8892827 10.1128/jb.178.21.6258-6265.1996 PMC178498

[B69] Qu Y Hyman P Harrah T Goldberg E In vivo bypass of chaperone by extended coiled-coil motif in T4 tail fiber J Bacteriol 2004 186 8363 8369 10.1128/JB.186.24.8363-8369.2004 15576786 PMC532435

[B70] Runnels JM Soltis D Hey T Snyder L Genetic and physiological studies of the role of the RNA ligase of bacteriophage T4 J Mol Biol 1982 154 273 286 10.1016/0022-2836(82)90064-X 7042981

[B71] Smith NL Taylor EJ Lindsay AM Charnock SJ Turkenburg JP Dodson EJ Davies GJ Black GW Structure of a group A streptococcal phage-encoded virulence factor reveals a catalytically active triple-stranded beta-helix Proc Natl Acad Sci USA 2005 102 17652 17657 10.1073/pnas.0504782102 16314578 PMC1308890

[B72] Olia AS Casjens S Cingolani G Structure of phage P22 cell envelope-penetrating needle Nat Struct Mol Biol 2007 14 1221 1226 10.1038/nsmb1317 18059287

[B73] Mitraki A Barge A Chroboczek J Andrieu JP Gagnon J Ruigrok RW Unfolding studies of human adenovirus type 2 fibre trimers. Evidence for a stable domain Eur J Biochem 1999 264 599 606 10.1046/j.1432-1327.1999.00683.x 10491109

[B74] Marusich EI Kurochkina LP Mesyanzhinov VV Chaperones in bacteriophage T4 assembly Biochemistry (Mosc) 1998 63 399 406 9556522

[B75] Bartual SG Garcia-Doval C Alonso J Schoehn G van Raaij MJ Two-chaperone assisted soluble expression and purification of the bacteriophage T4 long tail fibre protein gp37 Protein Expr Purif 2010 70 116 121 10.1016/j.pep.2009.11.005 19913618

[B76] Arisaka F Engel J Klump H Contraction and dissociation of the bacteriophage T4 tail sheath induced by heat and urea Prog Clin Biol Res 1981 64 365 379 7330053

[B77] Caspar DL Movement and self-control in protein assemblies. Quasi-equivalence revisited Biophys J 1980 32 103 138 10.1016/S0006-3495(80)84929-0 6894706 PMC1327271

[B78] Simon LD Anderson TF The infection of Escherichia coli by T2 and T4 bacteriophages as seen in the electron microscope. II. Structure and function of the baseplate Virology 1967 32 298 305 10.1016/0042-6822(67)90278-4 5337969

[B79] Simon LD Anderson TF The infection of Escherichia coli by T2 and T4 bacteriophages as seen in the electron microscope. I. Attachment and penetration Virology 1967 32 279 297 10.1016/0042-6822(67)90277-2 5337968

[B80] Kondou Y Kitazawa D Takeda S Tsuchiya Y Yamashita E Mizuguchi M Kawano K Tsukihara T Structure of the central hub of bacteriophage Mu baseplate determined by X-ray crystallography of gp44 J Mol Biol 2005 352 976 985 10.1016/j.jmb.2005.07.044 16125724

[B81] Fokine A Chipman PR Leiman PG Mesyanzhinov VV Rao VB Rossmann MG Molecular architecture of the prolate head of bacteriophage T4 Proc Natl Acad Sci USA 2004 101 6003 6008 10.1073/pnas.0400444101 15071181 PMC395913

[B82] Simpson AA Tao Y Leiman PG Badasso MO He Y Jardine PJ Olson NH Morais MC Grimes S Anderson DL Baker TS Rossmann MG Structure of the bacteriophage phi29 DNA packaging motor Nature 2000 408 745 750 10.1038/35047129 11130079 PMC4151180

[B83] Orlova EV Gowen B Droge A Stiege A Weise F Lurz R van Heel M Tavares P Structure of a viral DNA gatekeeper at 10 A resolution by cryo-electron microscopy Embo J 2003 22 1255 1262 10.1093/emboj/cdg123 12628918 PMC151052

[B84] Leiman PG Basler M Ramagopal UA Bonanno JB Sauder JM Pukatzki S Burley SK Almo SC Mekalanos JJ Type VI secretion apparatus and phage tail-associated protein complexes share a common evolutionary origin Proc Natl Acad Sci USA 2009 106 4154 4159 10.1073/pnas.0813360106 19251641 PMC2657435

[B85] Bingle LE Bailey CM Pallen MJ Type VI secretion: a beginner's guide Curr Opin Microbiol 2008 11 3 8 10.1016/j.mib.2008.01.006 18289922

[B86] Raskin DM Seshadri R Pukatzki SU Mekalanos JJ Bacterial genomics and pathogen evolution Cell 2006 124 703 714 10.1016/j.cell.2006.02.002 16497582

[B87] Pukatzki S Ma AT Revel AT Sturtevant D Mekalanos JJ Type VI secretion system translocates a phage tail spike-like protein into target cells where it cross-links actin Proc Natl Acad Sci USA 2007 104 15508 15513 10.1073/pnas.0706532104 17873062 PMC2000545

[B88] Mougous JD Cuff ME Raunser S Shen A Zhou M Gifford CA Goodman AL Joachimiak G Ordonez CL Lory S Walz T Joachimiak A Mekalanos JJ A virulence locus of Pseudomonas aeruginosa encodes a protein secretion apparatus Science 2006 312 1526 1530 10.1126/science.1128393 16763151 PMC2800167

[B89] Chenna R Sugawara H Koike T Lopez R Gibson TJ Higgins DG Thompson JD Multiple sequence alignment with the Clustal series of programs Nucl Acids Res 2003 31 3497 3500 10.1093/nar/gkg500 12824352 PMC168907

